# Carbon starvation of *Mycobacterium abscessus* induces a non-replicating state with extensive proteomic remodeling

**DOI:** 10.1128/msystems.00564-26

**Published:** 2026-09-23

**Authors:** Kaylyn L. Devlin, Gyanu Lamichhane, William C. Nelson, Vivian S. Lin, Kimberly E. Beatty

**Affiliations:** 1 Department of Chemical Physiology and Biochemistry, Oregon Health & Science University 6684, Portland, Oregon, USA; 2 Division of Infectious Diseases, School of Medicine, Johns Hopkins University 229385, Baltimore, Maryland, USA; 3 Biological Sciences Division, Pacific Northwest National Laboratory 536904, Richland, Washington, USA; 4 Institute of Biological Chemistry, Washington State University 57818, Pullman, Washington, USA; University of Southampton, Southampton, United Kingdom

**Keywords:** dormancy, proteomics, *Mycobacterium*, *Mycobacterium abscessus*, starvation, infectious disease, glycolipids, *Mycobacteroides*

## Abstract

*Mycobacterium abscessus* (*Mab*) is an opportunistic pathogen that can cause chronic, debilitating lung disease. *Mab* is intrinsically resistant to most antibiotics, making *Mab* infections challenging to manage and frequently incurable. During infection, *Mab* adapts to survive various stresses, including hypoxia and nutrient starvation. *In vitro*, these conditions drive *Mab* into a drug-tolerant, non-replicating state. Changes in the *Mab* proteome that result from entering a non-replicating state have been minimally described despite the clinical importance of this physiological state. Using *Mab* reference strain ATCC 19977, we collected proteomic data comparing replicating to non-replicating states using a carbon starvation (CS) model of persistence. We identified 2,251 proteins overall (45% proteome coverage), and 17% of these proteins were found in only one of the two conditions. A third of identified proteins were significantly changed in abundance, indicating an extensive proteomic response to CS. The response regulator DosR and many DosRS-responsive proteins were significantly more abundant under CS, suggesting that this response regulator plays a key role in CS-induced *Mab* persistence. Many aspects of cell wall biosynthesis were altered, including changes in glycolipid abundance under CS. Proteins involved in other key cellular processes such as secretion, oxidative phosphorylation, and nutrient metabolism were altered under CS. This proteomic analysis provides new insights and clarity into how the *Mab* proteome is regulated during non-replicating persistence, a key consideration for understanding *Mab* pathophysiology.

**IMPORTANCE:**

*Mycobacterium abscessus* (*Mab*) is a highly drug-resistant species of non-tuberculous mycobacteria (NTM). People most susceptible to pulmonary NTM infections are immunocompromised or have an existing lung disease, such as chronic obstructive pulmonary disease or cystic fibrosis. Infections with *Mab* cause chronic lung disease that is challenging to manage. Patients typically must take multiple antibiotics daily for at least a year. Despite aggressive treatment regimens, cure rates remain low. One reason for this is *Mab*’s ability to enter dormancy during chronic infections. The authors provide a first description of the proteomic remodeling that occurs in *Mab* in response to carbon starvation, which induces a non-replicating state. They report that a third of the proteome changes in response to carbon starvation, including pathways involved in cell envelope biosynthesis, energy production, and nutrient acquisition. The detailed description of these changes provides insights into the adaptations likely used by *Mab* to survive during infection.

## INTRODUCTION

*Mycobacterium abscessus* (*Mab*), also known as *Mycobacteroides abscessus* (1), is a non-tuberculous mycobacteria (NTM) that is ubiquitous within water- and soil-based environmental niches. *Mab* infections are most commonly acquired by inhalation and therefore establish within the lung, although *Mab* can also infect the skin and soft tissues following trauma (2). People with chronic lung conditions, such as bronchiectasis, chronic obstructive pulmonary disease, or cystic fibrosis, are most vulnerable to *Mab*-related disease. Infections are incredibly hard to cure due to intrinsic drug resistance, a fact that gives *Mab* the infamous designation as a “clinical nightmare” (3).

A hallmark of mycobacterial drug resistance is the ability of the pathogen to enter a non-replicating persistent state in response to host conditions, such as low pH, hypoxia, nutrient deprivation, and other factors (4). *Mab* is exposed to these stresses in the host within the granuloma (5, 6) and through biofilm formation (7, 8). Induction of a non-replicating state *in vitro* by modeling host conditions has demonstrated that persistent *Mab* is phenotypically drug tolerant (9–11), a factor that contributes to treatment failure. Efficacy testing of novel drugs against *Mab* is now encouraged to include persister assays to better predict clinical outcomes (4).

Despite the acknowledged clinical importance of persistent *Mab,* little is known about the proteomic characteristics of the non-replicating state. A thorough understanding of the pathophysiological adaptations of *Mab* to changing environments could improve treatment strategies or identify novel therapeutic targets. Several studies have investigated transcriptional changes in *Mab* persisters induced through exposure to hypoxia (12, 13), nutrient starvation (14), and growth in artificial sputum (13). However, transcription and translation are often decoupled (14), and it is the proteome that dictates overall physiology. To date, proteomic regulation by *Mab* persisters has been minimally described using models based on hypoxia (15), culture in phosphate-buffered saline (PBS; i.e., complete nutrient starvation), or potassium depletion (14).

In the current work, we sought to expand the understanding of *Mab* persistence and related proteomic adaptations. We used a new model of non-replicating persistence based on a previously described nutrient starvation model (9–11, 16, 17), which restricts carbon while maintaining inorganic nutrients. Our studies were completed in the reference strain, *M. abscessus* subspecies *abscessus* (ATCC 19977). We compared the *Mab* proteome under two physiologically distinct conditions: carbon-starved, non-replicating persistence and active replication. As described herein, we identified profound proteomic changes that occur in response to carbon starvation (CS), spanning key mycobacterial functions such as secretion, energy metabolism, virulence, and cell envelope biosynthesis and composition.

## RESULTS AND DISCUSSION

### A model for non-replicating persistence

Most *in vitro* models for *Mab* persistence have been adapted from those optimized for *Mycobacterium tuberculosis* (*Mtb*), such as the Wayne model (18) of hypoxia or the Betts model (19) of nutrient starvation. For example, Berube et al. (9) first adapted the Betts model for *Mab*, where bacteria were placed in PBS supplemented with tyloxapol for 4 days. They found that nutrient-starved *Mab* retained susceptibility to amikacin but was resistant to most other drugs. Yam and coworkers (10) also used a PBS-based nutrient starvation model, reporting suspension of growth and maintenance of viability over 10 days, along with high tolerance to a range of antibiotics in these non-replicating persisters.

However, we observed an initial decrease in *Mab* colony-forming units (CFUs) when cultured in PBS (20). As a result, we based our non-replicating *Mab* model on the Hung model (16) of carbon starvation, which has been used in numerous studies of *Mtb* (17, 21–23). *Mab* was cultured for 3 days in Middlebrook 7H9 broth supplemented with tyloxapol only (CS), constituting starvation of carbon with the maintenance of inorganic compounds. CS halted growth by day 2 and maintained viability through day 8 (study termination), without the initial apparent cell death observed with PBS-only culture (20). We therefore used the CS-induced non-replicating state as a model of *Mab* persistence for our proteomic analyses. In the current study, proteomics was performed on lysates collected at the 72-h (3-day) time point. Lipid analysis was performed at both the 3- and 7-day time points.

### Global proteomics analysis

**Fig 1:**
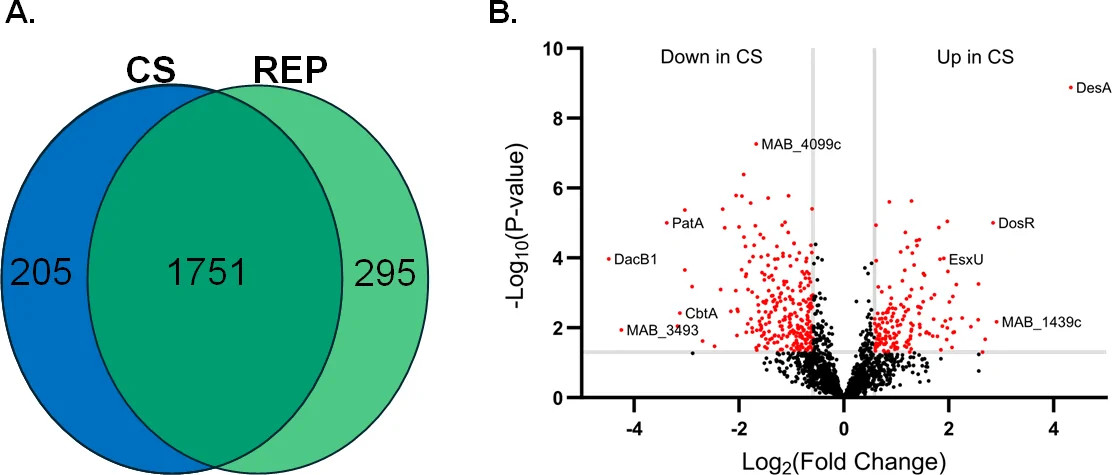
Comparison of proteins identified in CS versus REP conditions. (**A**) Venn diagram of proteins identified in *n* ≥ 3 samples in CS or REP conditions. (**B**) Volcano plot of proteins found in CS and REP conditions. Red dots highlight proteins significantly different in abundance in CS versus REP, with *P* ≤ 0.05 (ANOVA), and a log_2_ fold change ≥0.585 (up in CS) or ≤−0.585 (down in CS), corresponding to a fold change of >1.5. Proteins identified in only one group (*n* ≥ 3 in group A and *n* ≤ 1 in group B) were not included in the volcano plot due to the inability to calculate a fold change.

Global proteomic profiles were generated for *Mab* grown under CS for 72 h and for actively growing *Mab* from nutrient-complete cultures (REP) using mass spectrometry-based proteomics. To ensure rigor, we only considered proteins that were identified in three or more of the six biological replicates of either group (REP or CS). Overall, we identified 2,251 proteins out of the 4,949 annotated in *Mab* (45% proteome coverage; Table S1). A similar number of proteins was identified in REP (2,046) and CS (1,956) conditions, and most proteins (1,751) were identified in both (Fig. 1).

We used spectral intensities as an estimate of protein abundance to identify differentially expressed proteins (DEPs) between growth conditions. We defined DEPs as proteins changed in either abundance or presence (Table S2). Abundance was analyzed (ANOVA, *P* ≤ 0.05, fold change [FC] >1.5) when the protein was identified in both groups (*n* ≥ 2 out of 6) (Fig. 1B). A significant change in presence was analyzed (G-test, *P* ≤ 0.05) when the protein was absent in one group (*n* ≥ 3 in group A and *n* ≤ 1 in group B). In subsequent heat maps, the lack of a mean intensity value and a FC for proteins absent in one group are denoted by a white and gray box, respectively.

We identified 288 DEPs in CS, with 125 present only in CS (*n* ≤ 1 in REP). Conversely, there were 463 DEPs in REP, with 194 present only in REP (*n* ≤ 1 in CS). Overall, 33% of proteins in our data set were significantly changed in response to CS, indicating substantial proteomic regulation in persistent *Mab*.

Functional classification of DEPs in *Mab* is challenging because the *Mab* proteome remains poorly annotated, and 40% of proteins have no known function. We used *Mtb* orthology to enrich *Mab* protein classification. A multistep analysis was performed to identify orthologs between *Mab* 19977 and *Mtb* H37Rv (Table S3). Integration of reciprocal best hits, OrthoFinder (24), Markov clustering, MMSEQS2 clustering (25), and consideration of gene synteny were performed to optimally identify one-to-one gene mappings across organisms. In agreement with similar analyses (26–28), less than 50% of *Mab* genes (2,334) had a high-confidence ortholog in *Mtb* (Fig. S1). No ortholog was identified for 2,566 *Mab* genes. Using UniProt (29) annotations and *Mtb* orthology, we assessed specific CS-related proteomic changes across key cellular and pathogenic processes including intra- and extracellular signaling, cell wall biosynthesis, and energy and nutrient metabolism (Table S4).

### Two-component signaling systems and DosRS

**Fig 2:**
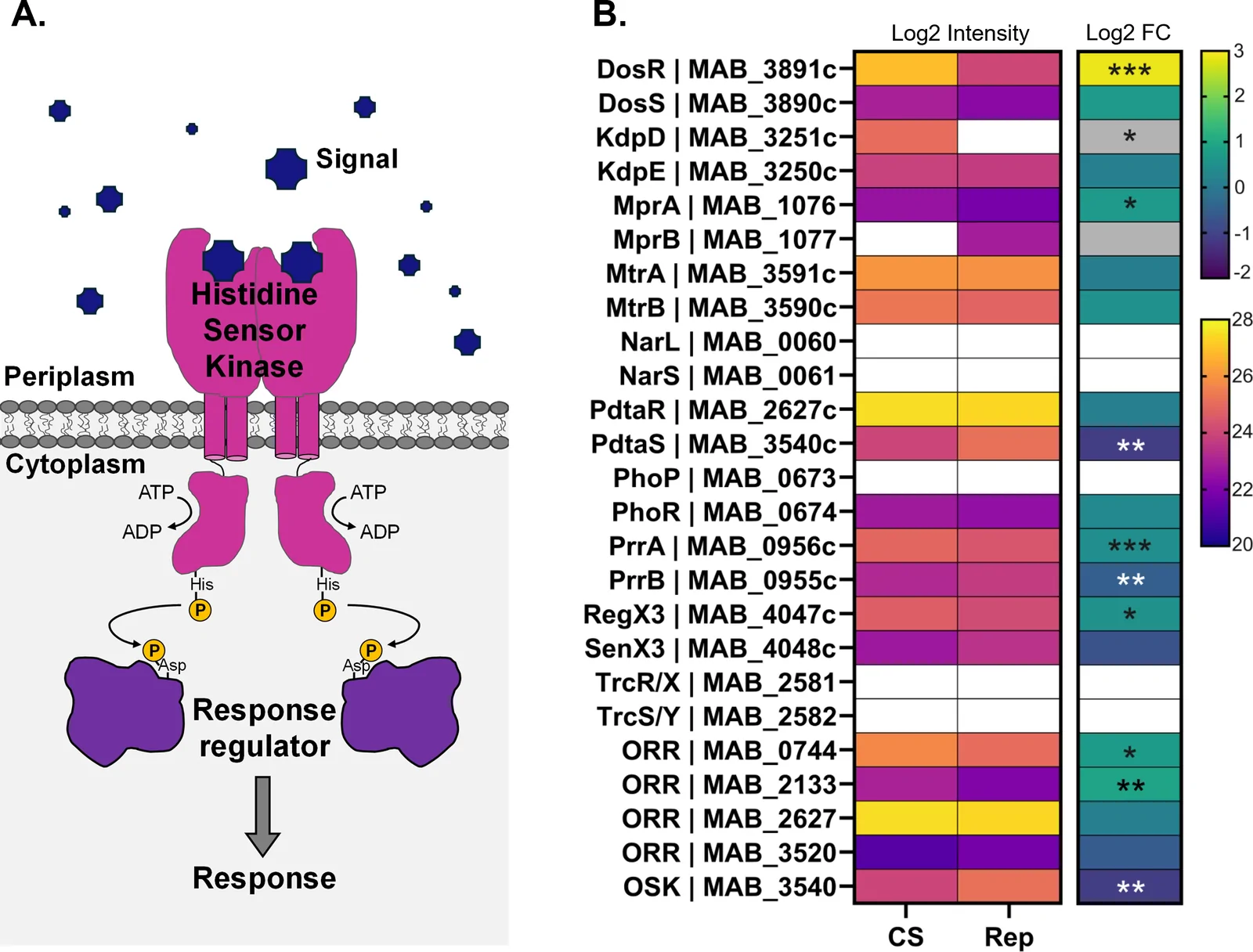
Two-component signaling systems are altered by carbon starvation. (**A**) Schematic of a two-component signaling system, which uses a histidine sensor kinase and a response regulator to regulate gene expression in response to a signal from the environment. (**B**) Heat map of mean log2 intensities (left map, warm scale) and corresponding log2 fold change (right map, cool scale) of TCSS proteins in CS versus REP (*n* = 6) groups. The locus identity is provided for each protein. In this and subsequent heat maps, a gray box indicates that a protein was present in only one group (group A: *n* ≥ 3, Group B: *n* ≤ 1), and a white box indicates the absence of value. In all heat maps, asterisks denote the significance of the difference in presence (G-test) or mean intensity (ANOVA) between CS and REP (*, *P* ≤ 0.05; **, *P* ≤ 0.01; ***, *P* ≤ 0.001). ORR: orphan response regulator. OSK: orphan sensor kinase.

Mycobacterial two-component signal transduction systems (TCSS) regulate major cellular processes such as growth, persistence, and virulence in response to environmental signals (30). Those signals can include low pH, oxidative stress, metals, nutrients, antibiotics, and other factors. TCSS comprise a pair of proteins, including a periplasm-facing histidine sensor kinase that confers a signal to a cytoplasmic response regulator (Fig. 2). Activated response regulators bind DNA to induce transcriptional changes.

*Mab* encodes at least ten TCSS and five orphan components (regulators or kinases). We observed significant changes in abundance of nearly half of these proteins in response to CS (Fig. 2B). The largest change was in DosRS. Prior studies showed that DosRS is required for growth, survival, and adaptation to hypoxia (12, 31). We found that the response regulator, DosR (locus identity: MAB_3891c), was 7.2-fold more abundant in CS samples compared to REP. The abundance of the sensor histidine kinase DosS (MAB_3890c) was comparable between CS and REP. Our findings are in accordance with other persistence and stress models, as transcripts of these regulators were found to be elevated in response to hypoxia (*dosR* and *dosS*) (12, 13) and intracellular survival within macrophages (*dosR* only) (32). However, a recent study looking at *Mab* proteomic changes in response to total nutrient starvation (culture in PBS) did not find DosR or DosS altered at the protein level compared to active replication (14). Therefore, our results provide the first evidence that DosRS is used by *Mab* to respond to carbon starvation.

We compared our dataset to DosRS-regulated *Mab* genes identified as transcriptionally upregulated in hypoxia. We limited our comparison to the ten most induced DosRS-regulated genes identified by either Simcox et al. (12) or Belardinelli et al. (31) for a total of 11 proteins (Table S4). Eight were identified in our data set, seven of which were significantly more abundant in CS (>4-fold; *P* < 0.0002). These proteins included a fatty acid desaturase DesA (MAB_3354), adenylate kinase (MAB_3902c), NADPH nitroreductase (MAB_3903), cytochrome c oxidase subunit (MAB_1042c), two universal stress protein A (UspA) domain-containing proteins (MAB_3904 and MAB_2489), and an unannotated protein (MAB_3937). The remaining identified protein within the comparison set, a nitric oxide dioxygenase (MAB_3133c), was only found in REP (not significant; *P* = 0.071). Overall, our proteomic data support that DosRS serves as a key regulatory mechanism for responding to carbon starvation in *Mab*, mirroring many of the responses observed under hypoxia.

There were changes in several uncharacterized *Mab* TCSS between CS and REP samples. We identified the sensor kinase KpdD (MAB_3251c) in CS alone, while its paired response regulator KpdE (MAB_3250c) was unchanged between CS and REP. The orthologous *Mtb* TCSS are induced under nutrient stress (19). PdtaR/S is uncharacterized in *Mab*, but we found that the sensor kinase PdtaS (MAB_3540c) was 2.2-fold less abundant in CS. There were two recent reports characterizing *Mtb*’s PdtaR/S. One found that it plays a role in virulence and persistence (33). The second found that PdtaR/S senses heme and regulates oxidative stress responses (34). Next, SenX3/RegX3 regulates virulence in *Mtb*; the *Mab* RegX3 (MAB_4047c) response regulator was slightly, but significantly, more abundant in CS (1.4-fold). The function of MprA/B in *Mab* is unknown, but these proteins are necessary for persistence in *Mtb* (35, 36). MprA was 1.6-fold more abundant in CS, although its cognate kinase MprB was only identified in REP.

TCSS have emerged as an important area of *Mab* research, with several studies published since 2023. For example, MtrA/B was recently characterized to impact drug resistance, virulence, and cell division (37). We identified MtrA/B, but its abundance was not significantly different between REP and CS. The orphan response regulators GlnR (MAB_0744) and NnaR (MAB_3520c) (38), which mediate nitrogen metabolism, have recently been characterized. GlnR, which also facilitates biofilm formation (39), was 1.6-fold more abundant in CS compared to REP, while NnaR was not significantly different. Finally, PhoP/R mediates the response to low pH (40); PhoR was identified but unchanged.

### Secretion systems

**Fig 3:**
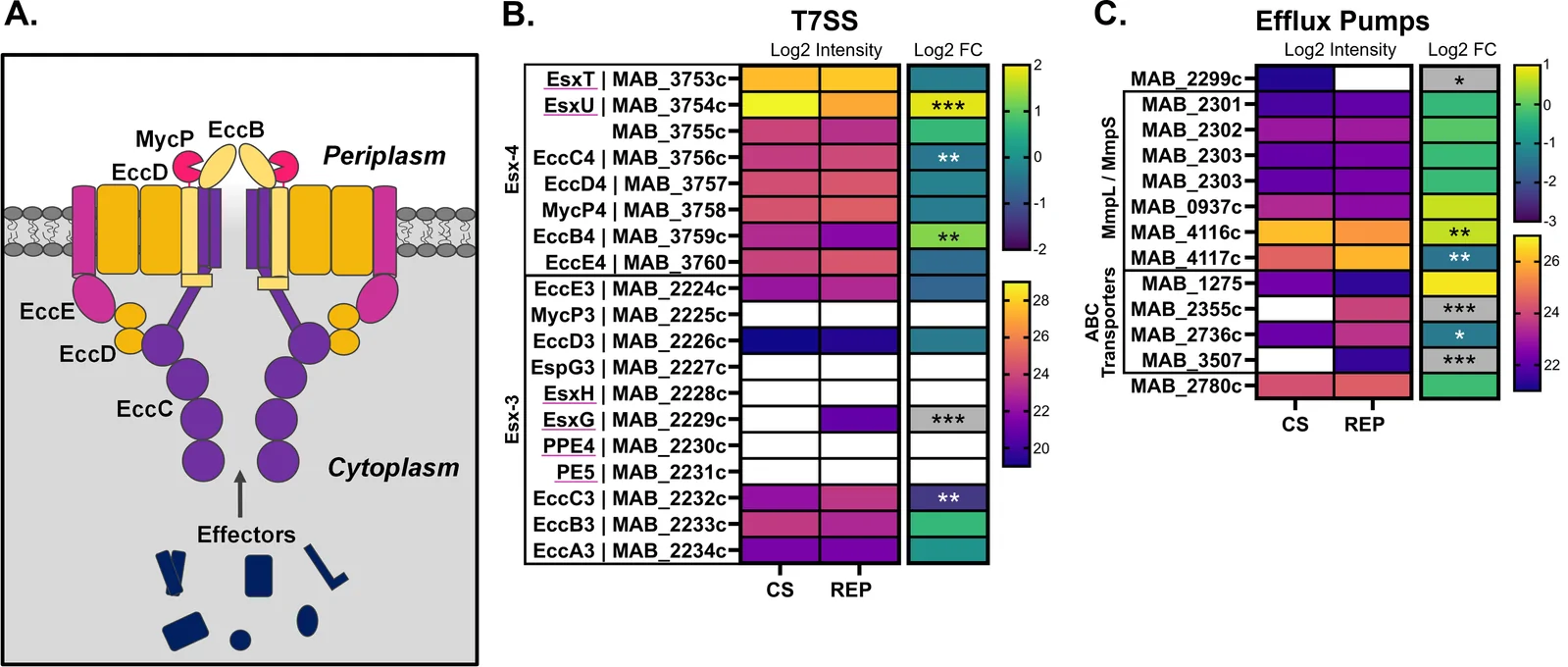
Organization and regulation of *Mab* secretion systems under CS. (**A**) Functional organization of mycobacterial T7SS pore-forming components. (**B and C**) Heat maps of mean log2 intensities (left map, warm scale) and corresponding log2 fold change (right map, cool scale) of T7SS proteins (**B**) and efflux pumps (**C**) in CS versus REP. Underlined proteins in (**B**) are substrates of Esx3 or Esx4.

Type VII secretion systems (T7SS) are used by mycobacteria to export proteins and nutrients across the cell envelope (Fig. 3). T7SS are linked to pathogenesis, immune system evasion, and survival in the host (41, 42). While *Mtb* encodes five T7SS, *Mab* encodes only two: Esx3 and Esx4. Esx3 (MAB_2224c–MAB_2234c) plays a role in growth, persistence, and pathological responses during infection (13, 43). We identified five components of the Esx3 core complex, and EccC3 (MAB_2232c) was 2.6-fold less abundant in CS (Fig. 3B). We identified an Esx3 substrate, EsxG (MAB_2229c), only in REP samples. Both EccC3 and EsxG were recently found to be reduced at the protein level in response to total nutrient starvation in *Mab*, similar to our findings (14).

Esx4 contributes to intracellular survival by blocking phagosomal acidification and inducing phagosomal rupture during infection (44). All Esx4 components (MAB_3753c–MAB_3760) were identified in both conditions (Fig. 3B). We found that a key structural component, EccB4 (MAB_3759c), was 2.4-fold more abundant in CS. Additionally, Esx4’s substrate EsxU (MAB_3754c) was 3.6-fold more abundant in CS. A recent study showed that heterodimeric EsxU/T is upregulated during infection, where it is involved in phagosomal membrane rupture and reducing virulence (45). Taken together, our data suggest that Esx4 may have an important role in *Mab* under conditions of CS, while Esx3 may be more relevant during active growth.

The induction of efflux pumps is hypothesized to contribute to drug resistance in mycobacterial pathogens (46). Several recent studies demonstrated that *Mab* upregulates efflux pumps in response to antibiotic treatment (47–51). We examined our data and found six significant changes in both efflux pumps and their transcriptional regulators (Fig. 3C). For example, MAB_2299c, a transcriptional regulator associated with clofazimine and bedaquiline resistance (50), was identified only in CS. The corresponding membrane proteins, MAB_2301 and MAB_2302, were unchanged. An efflux pump linked to macrolide resistance (49), MAB_2355c, was found only in REP. Finally, the ABC transporter MAB_3507 was found only in REP.

### The mycobacterial cell envelope

#### Peptidoglycan biosynthesis and degradation

**Fig 4:**
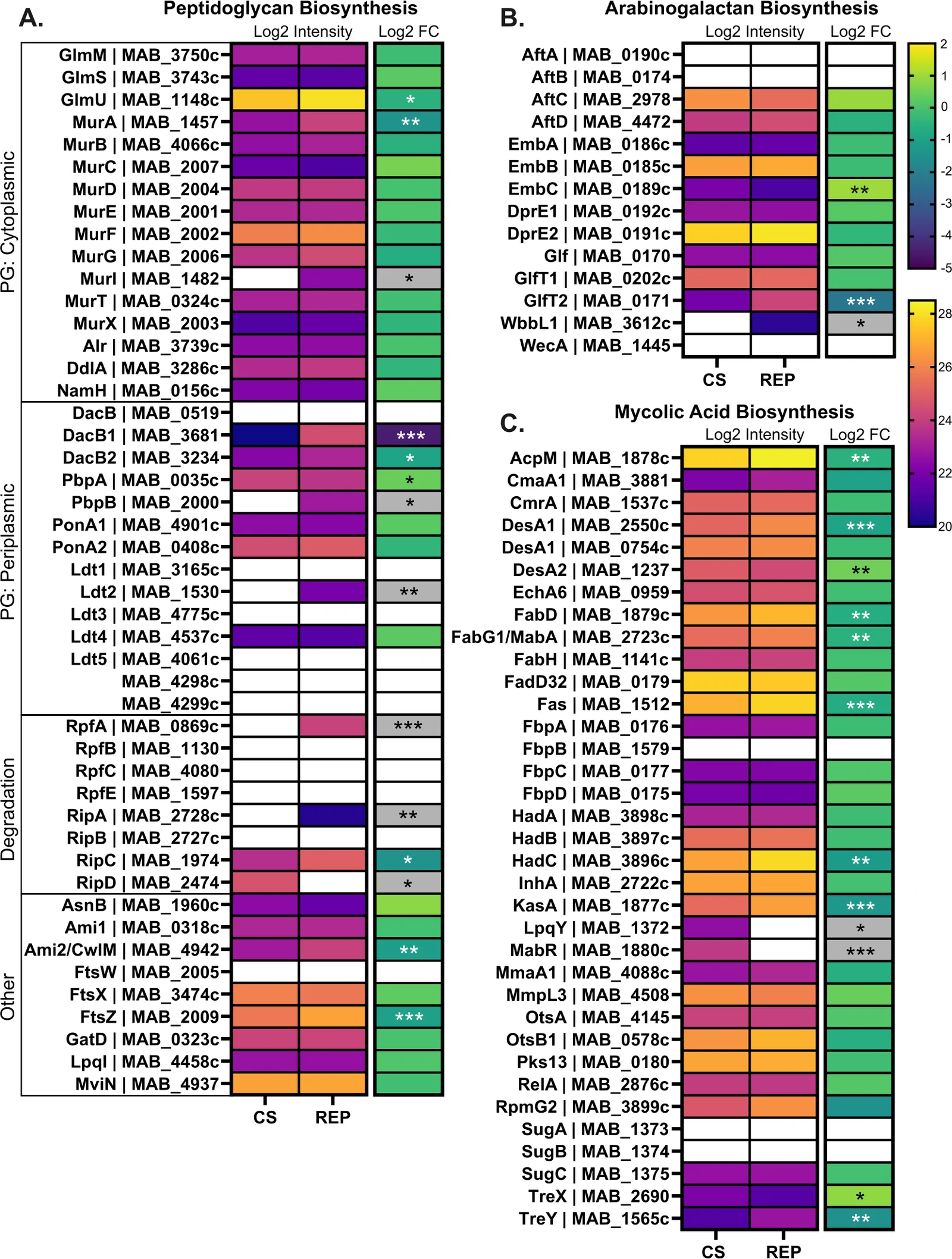
Regulation of cell envelope biosynthesis under CS. Heat maps of mean log2 intensities (left map, warm scale) and corresponding log2 fold change (right map, cool scale) in CS versus REP (*n* = 6) groups of proteins involved in peptidoglycan (**A**), arabinogalactan (**B**), and mycolic acid (**C**) biosynthesis. Scale bars are applicable across all corresponding maps in panels A–C. In panel C, DesA1 (MAB_3354) was excluded from the heat map due to the large fold change skewing color mapping.

Peptidoglycan (PG) is a massive polymer that encapsulates bacterial cells and serves as their exoskeleton. In mycobacteria, it is comprised of a glycan backbone of N-acetylglucosamine and N-acetylmuramic acid attached to peptide stems, which are highly cross-linked. PG biosynthesis commences in the cytoplasm with synthesis of UDP-N-acetylglucosamine attached to the peptide stem. These steps are catalyzed by GlmS (MAB_3743), GlmM (MAB_3750c), GlmU (MAB_1148c), and MurA/B/C/D/E/F/G/I. NamH (MAB_0156c), alanine racemase (Alr; MAB_3739c), and D-Ala-D-Ala ligase (DdlA; MAB_3286c) also play a role (52). All of these proteins were identified in both CS and REP groups (Fig. 4A). Two were less abundant in CS (GlmU and MurA), and MurI was only identified in REP conditions. Rojony et al. (15) reported that MurC/D/E/F/G/X were upregulated in hypoxia and biofilm conditions, but we found no significant differences.

The remaining biosynthetic steps take place in the periplasm, where carboxypeptidases trim the peptide stems and transpeptidases cross-link them. *Mab*’s PG contains two distinct types of cross-links. The first type is the classically described D-Ala-mDap (4→3) cross-link, which is formed by D,D-transpeptidases (DDTs). These enzymes are also called penicillin-binding proteins (PBPs). We identified four DDTs in our dataset: PbpA (MAB_0035c), PbpB (MAB_2000), PonA1 (MAB_4901c), and PonA2 (MAB_0408c). PbpA was slightly more abundant in CS (1.3-fold), and PbpB, an essential enzyme (27), was only identified in REP samples. Mycobacterial PG also has mDap-mDap (3→3) “non-classical” cross-links. The latter predominate in *Mab* and are formed by L,D-transpeptidases (LDTs) using a tetrapeptide substrate made by carboxypeptidases (53). The carboxypeptidases DacB1 (MAB_3681) and DacB2 (MAB_3234) were identified in our dataset. Both were substantially less abundant in CS samples, with DacB1 being among the most CS-responsive proteins (22.3-fold less abundant). There are seven LDT-domain-containing proteins in the *Mab* proteome (i.e., Ldt1-5, MAB_4298c, and MAB_4299c) (20). We found Ldt4 (MAB_4537c) in both CS and REP, while Ldt2 (MAB_1530) was only found in REP samples. Overall, our data demonstrate that many enzymes associated with PG biosynthesis are less abundant in CS, and distinct PG-modifying enzymes are used in replicating versus non-replicating states. We recently described the results from activity-based protein profiling in REP and CS *Mab*, in which β-lactam antibiotic targets were identified using a biotinylated meropenem (20). We determined that carboxypeptidases, DDTs, and LDTs were active in both growth conditions, suggesting that these proteins remain druggable in CS despite reduced abundance.

There are various PG-degrading enzymes encoded by *Mab*, including the resuscitation promotion factors (RpfA–E) and endopeptidases (RipA–D). In *Mtb*, RpfA is required for the recovery of growth after dormancy (54), and RipA is localized to the septa of dividing cells (55). Our data indicate that these degraders are regulated in response to CS. We found RipD in CS alone, while RpfA and RipA were in REP alone. RipC was less abundant in CS. Grigorov et al. (14) reported similar results, with RpfA and RipA proteins reduced in total nutrient-starved *Mab*. The absence of RpfA and RipA in CS was therefore expected; however, it served as confirmation that our CS conditions induced a non-replicating state.

#### Arabinogalactan biosynthesis

The next layer of the cell wall is arabinogalactan (AG), which is covalently linked to both PG and mycolic acids (MA). AG comprises branched arabinose chains attached to galactan. The galactan chain is made by WbbL1 (MAB_3612c), GlfT1 (MAB_0202c), GlfT2 (MAB_0170), and likely other enzymes not yet annotated in *Mab*. WbbL1 was only found in REP samples, and GlfT2 was significantly less abundant in CS (4.8-fold) (Fig. 4B). Since these enzymes are involved in early AG biosynthetic steps, it is possible that AG production is reduced in CS.

In the periplasm, arabinose is added to the galactan portion by various arabinosyltransferases, including AftA-D. AftC and AftD were not different between CS and REP samples. The targets of ethambutol are the arabinosyltransferases EmbA/B/C (56). EmbC (MAB_0189c) was 1.8-fold more abundant in CS, while EmbA (MAB_0186c) and EmbB (MAB_0185c) were unchanged. DprE1 (MAB_0192c) and DprE2 (MAB_0191c) also play a role in AG biosynthesis; they were unchanged between CS and REP.

#### Mycolic acid biosynthesis

A distinguishing feature of the mycobacterial cell wall is the outer mycomembrane containing long-chain mycolic acids (MAs), including trehalose monomycolate and trehalose dimycolate. We estimate that 20% of enzymes involved in MA biosynthesis, modification, and transport were less abundant in CS (Fig. 4C). The key fatty acid synthesis enzyme Fas (MAB_1512) was 1.7-fold less abundant in CS. Next, fatty acids are extended by the FAS-II complex of enzymes. MabR (MAB_1880c) is a predicted transcriptional regulator of the *fasII* operon based on its orthology to *Mtb*’s MabR (Rv2242) (57). Our proteomics data suggest that it could play a similar role in *Mab*, as several FAS-II enzymes were less abundant in CS, including KasA (MAB_1877c; 2.5-fold), HadC (MAB_3896c; 2.3-fold), and MabA/FabG1 (MAB_2723c; 1.5-fold). Notably, a reduction in KasA protein levels is involved in biofilm formation in *Mycobacterium smegmatis* (58). InhA (MAB_2722), a target of isoniazid, was unchanged between CS and REP. In *Mtb*, there are two pathways that make trehalose, OtsA/OtsB1 and TreX-TreY-TreZ (59). We found that OtsA/OtsB1 were unchanged between CS and REP, while TreX (MAB_2690) was 1.8-fold more abundant in CS than REP. Taken together, these findings suggest that the as-yet uncharacterized TreX-TreY-TreZ pathway might play a more important role than OtsA/OtsB1 under nutrient-limited conditions in *Mab*. An ortholog of TreZ has not yet been identified in *Mab*.

Attachment of MA to AG is mediated by the antigen 85 complex (FbpA-D). We identified three of the four components of this complex as unchanged between CS and REP. The mycolic acid desaturase DesA1 has several putative orthologs in *Mab*, including MAB_3354, MAB_2550c, and MAB_0754c. Interestingly, two of these were significantly changed in opposite directions under CS. MAB_3354 was substantially more abundant in CS (20-fold, Table S4, excluded from heat map), while MAB_2550c was 1.9-fold less abundant in CS. DesA2 (MAB_1237) was 1.4-fold less abundant in CS. LpqY (MAB_1372), which is involved in sugar transport and trehalose recycling in *Mtb* (60), was found only in CS. To summarize, our data suggest that *Mab* reduces MA biosynthesis in response to carbon starvation, mirroring how *Mtb* responds to nutrient starvation (17, 60, 61).

#### PIM biosynthesis

**Fig 5:**
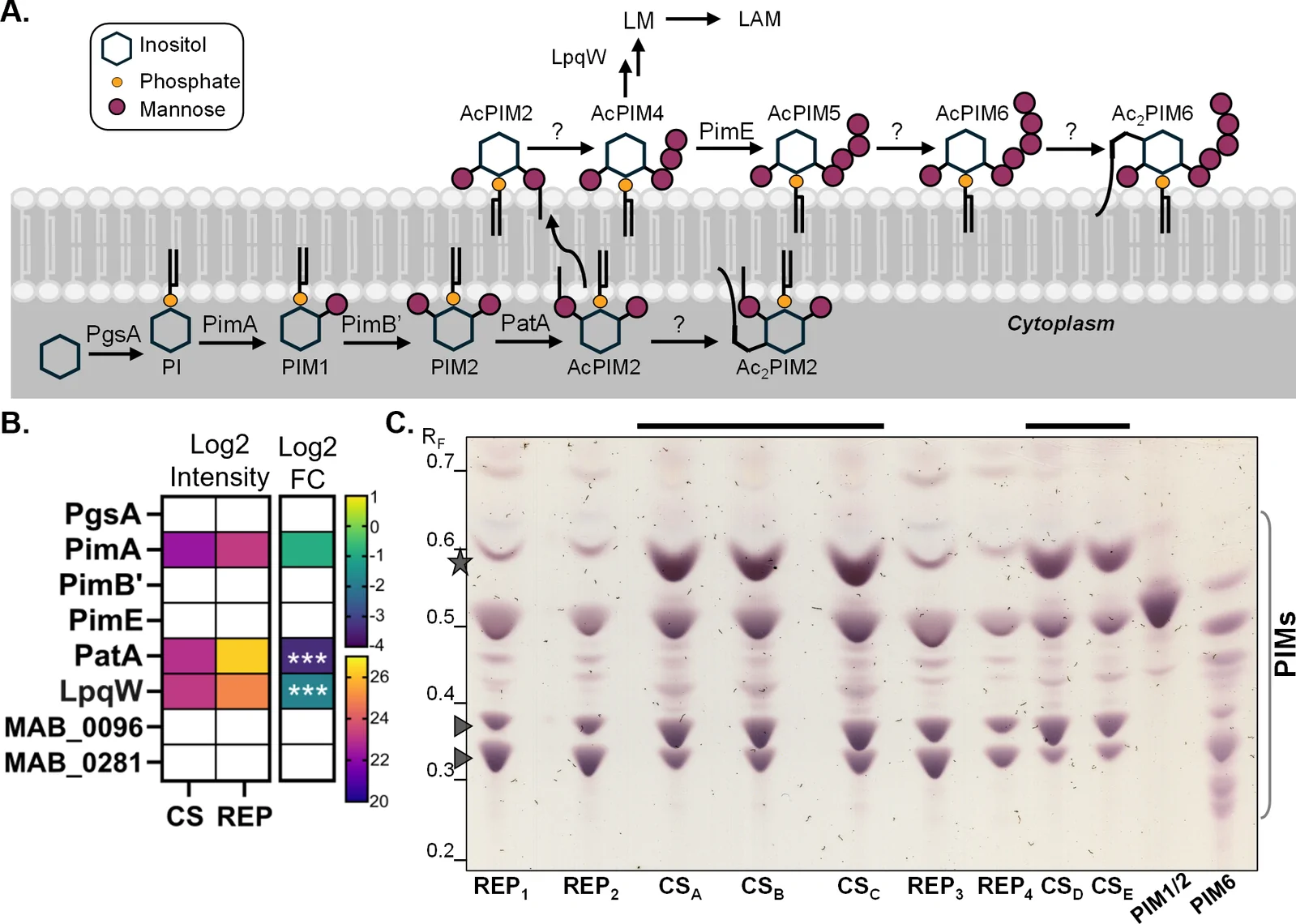
PIM biosynthesis is altered under CS. (**A**) Schematic of the biosynthesis of mycobacterial PIMs. In *Mab*, the predominant PIMs are acylated PIM2 and PIM6. (**B**) Heat map of mean log2 intensities (left map, warm scale) and corresponding log2 fold change (right map, cool scale) in CS versus REP groups (*n* = 6) of proteins involved in PIM biosynthesis. (**C**) Thin layer chromatography (TLC) separation of normalized polar lipid extracts from cultures of *Mab*. PIMs were resolved in chloroform, methanol, ammonia, 0.1 M ammonium acetate, and water (vol/vol; 180:140:9:9:23) and stained with orcinol. Putative PIMs are indicated by a bracket. A PIM species at R_F_ ~0.6 with increased abundance in CS samples is indicated with a star. Two species (R_F_ = 0.3–0.4) changed in relative abundance are indicated by arrowheads. PIM1/2 and PIM6 standards purified from *M. tuberculosis* H37Rv were obtained from BEI Resources.

Phosphatidylinositol mannosides (PIMs) are abundant plasma membrane glycolipids in mycobacteria (62). Most mycobacterial PIMs contain either two (PIM2) or six (PIM6) mannose residues (Fig. 5) (63). We examined the PIM biosynthetic machinery in our proteomics dataset and found that the key PIM acyltransferase, PatA (MAB_2895c), was 10.4-fold less abundant in CS (Fig. 5B). Other biosynthetic components were not found (PimE and PimB′) or unchanged (PimA). LpqW (MAB_1315), an essential lipoprotein (27), was 3.5-fold less abundant in CS. Studies of the ortholog from *M. smegmatis* suggest that LpqW regulates PIM levels relative to lipoarabinomannans (64, 65).

We sought to further describe the consequences of CS on PIMs. To do so, we analyzed the relative abundance of PIMs isolated from CS and REP cultures of *Mab*. Extracted polar lipids were resolved by TLC and stained with orcinol (Fig. 5C). Loading was normalized for each sample based on the culture’s optical density. Purified *Mtb* PIM1/2 and PIM6 were included as migration standards (BEI Resources); *Mab* PIM standards were unavailable. We observed numerous resolved bands most likely corresponding to PIM2 and PIM6 species, based on their migration (i.e., retention factor [R_F_]) and orcinol staining (63, 66). Notably, we observed an increased abundance in CS of a species just below R_F_ = 0.6. We have not identified the PIM species, but the migration suggests that it could be a di-acylated PIM2 (Ac_2_PIM2). This finding would be consistent with a previous study by Morita and coworkers (63), which demonstrated that PIM acylation is a stress response in mycobacteria, including *Mab*. We additionally observed a change in relative intensity for two lower bands (R_F_ = 0.32 and 0.38), which likely correspond to a highly mannosylated PIM species (i.e., PIM6).

#### Glycopeptidolipid biosynthesis

**Fig 6:**
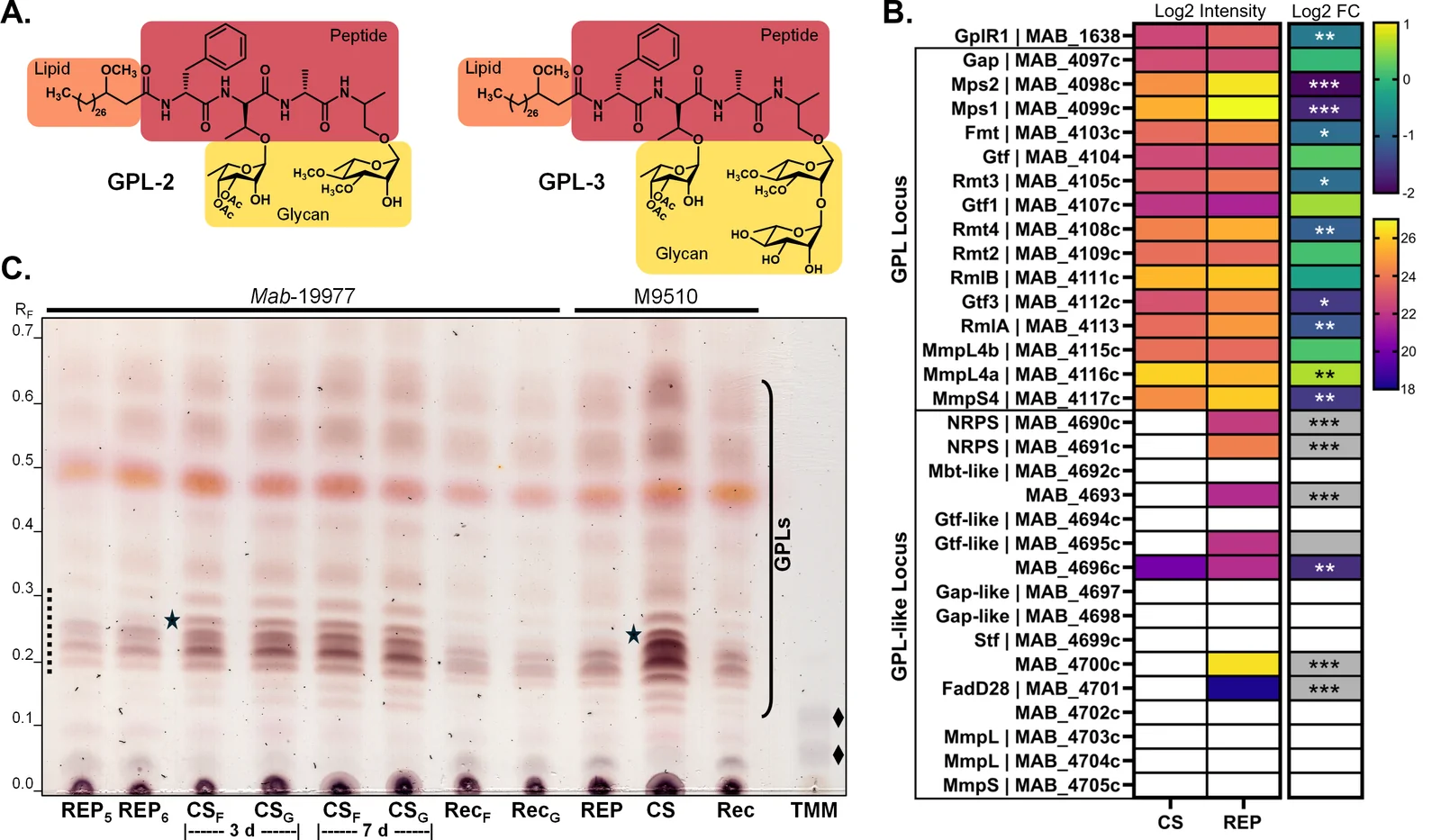
GPL biosynthesis is altered under CS. (**A**) Structures of two GPLs made by *Mab*, GPL-2 and GPL-3. (**B**) Heat map of mean log2 intensities (left map, warm scale) and corresponding log2 fold change (right map, cool scale) in CS versus REP groups of proteins involved in GPL biosynthesis. NRPS = non-ribosomal peptide synthase. (**C**) TLC analysis of normalized total lipid extracts from *Mab* 19977 and a clinical isolate, M9510. Total lipids were resolved in chloroform/methanol/water vol/vol; 90:10:1), and glycolipids (e.g., GPLs) were visualized by orcinol staining. Putative GPLs are indicated by a bracket, with the region (R_F_ = ~0.2–0.3) marked by a dashed line predicted to correspond to GPL-3. Black stars (R_F_ = 0.26) indicate a species detected only in CS. Trehalose monomycolate (TMM) purified from *M. tuberculosis* H37Rv was obtained from BEI Resources and included as a reference marker (diamonds).

Glycopeptidolipids (GPLs) are abundant lipids on the outer cell envelope of some species of mycobacteria, including *Mab* (67). *Mab* biosynthesizes both di-glycosylated GPL-2 and tri-glycosylated GPL-3 (Fig. 6). In *Mab*, GPLs are linked to biofilm production, morphotype, and pathogenicity (68). Generally, the presence of GPLs is associated with a “smooth” morphotype, while the absence is associated with a “rough” morphotype. The rough morphotype is considered clinically relevant and is associated with late-stage, severe disease (69, 70). Genomic analysis of clinical isolates demonstrated that a rough morphology can result from mutations in genes found in the *gpl* locus, including *mps1*, *mps2*, *gap*, *mmpL4*, and *mmpS4* (70, 71). There is evidence that *Mab* can modulate GPL levels reversibly, although factors that cause these changes are largely unknown (72–74). Prior work by Boutte and coworkers (75) found that GPLs were unchanged by growth in rich Middlebrook 7H9 culture medium compared to either Hartmans-de Bont minimal medium or artificial cystic fibrosis sputum medium. In contrast, Jackson and coworkers observed an increase in the ratio of GPL-3 to GPL-2 and production of new GPL species in response to mucin exposure (74), including in synthetic cystic fibrosis sputum medium and cystic fibrosis sputum.

Factors that dynamically impact GPL production in response to the environment are unknown. We therefore analyzed our proteomics data for changes associated with CS (Fig. 6B). First, we found that the transcription factor GplR1 (MAB_1638) was 1.7-fold less abundant in CS. Recent work demonstrated that GplR1 is required for GPL production (76) and influences the expression of both the *gpl* locus (MAB_4097c—MAB_4117c) and the *gpl*-like locus (MAB_4689c—MAB_4705c). We found that most of the proteins encoded in these two loci were less abundant in CS. This evidence supports that CS impacts GPL biosynthesis.

Based on our proteomics data, we hypothesized that the GPL content would change between CS and REP cultures. We compared total lipid extracts (Fig. 6C) and polar lipid extracts (Fig. S2) isolated from REP and CS cultures of *Mab*. We additionally collected lipids from recovered (Rec) *Mab*, which was obtained by diluting and culturing carbon-starved *Mab* for 1 day in complete, nutrient-rich medium. Extracts were analyzed by TLC, with loading normalized based on the culture’s optical density. We included *Mtb* trehalose monomycolate (TMM) as a migration standard, although we did not analyze mycolic acids. Instead, we focused on the GPLs, which had higher overall levels in CS. A set of bands that migrate and stain like GPL-3 (77, 78) was noticeably more abundant in CS at both 3- and 7-day time points (Fig. 6C). We also observed a species associated with CS only (R_F_ = 0.26). The changes in GPL content were reversible, with reversion to a profile like REP in Rec samples. We additionally analyzed total lipids isolated from a clinical strain, *Mab* M9510 (subspecies *massiliense*) (79). We observed similar changes in GPLs in this strain cultured under CS, suggesting that this response to CS is conserved across subspecies. The increase in GPLs under CS was an unexpected result because most of the GPL biosynthetic enzymes were less abundant in CS (Fig. 6B). We speculate that there could be unidentified GPL-degradative enzymes that are absent in CS. There are other possibilities, such as post-translational regulation of GPL biosynthetic enzyme activity. Overall, our results suggest that CS results in a reversible enrichment of GPLs in *Mab*, a novel finding that warrants further exploration.

### Energy metabolism

#### Oxidative phosphorylation

**Fig 7:**
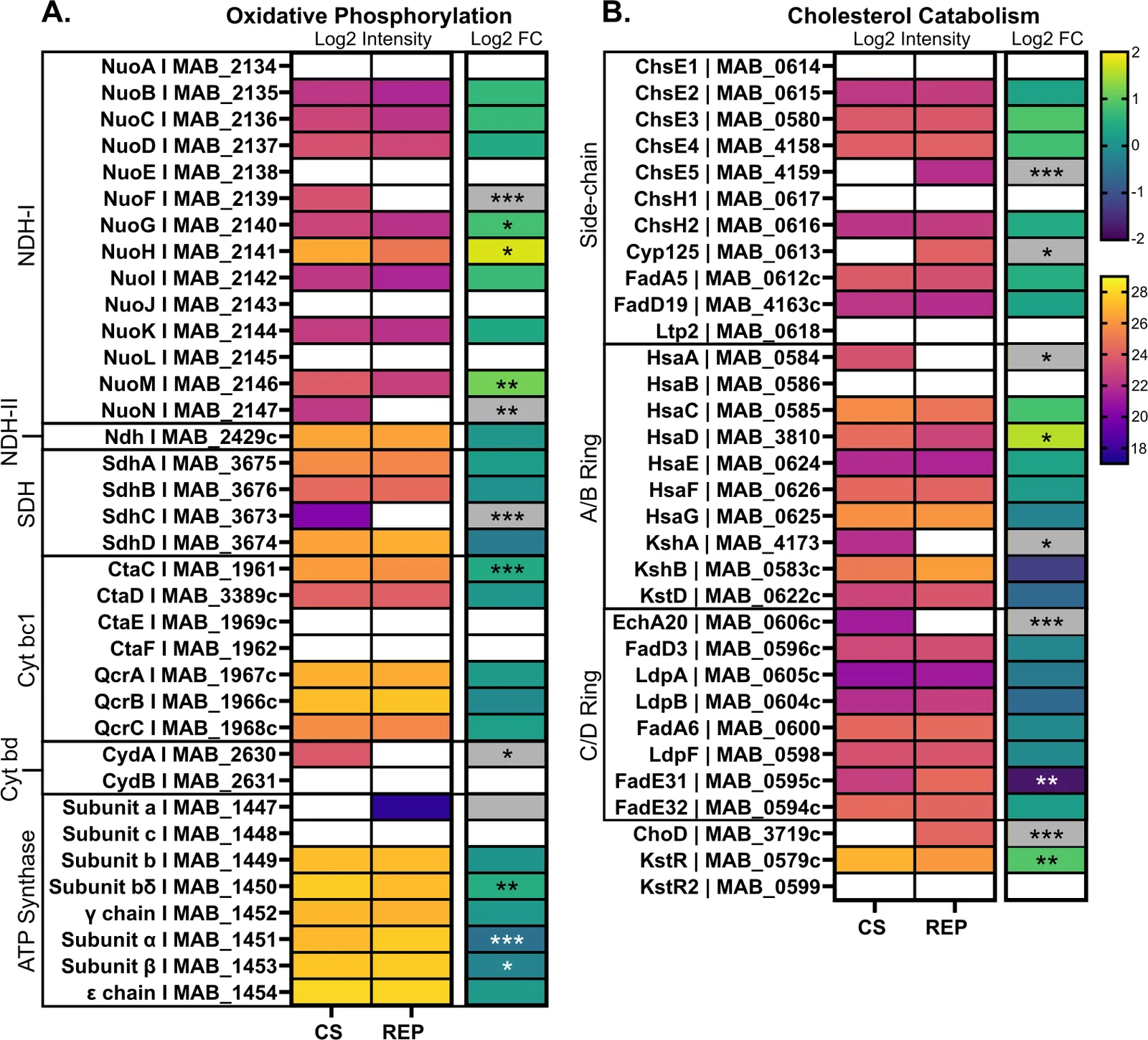
Regulation of energy and cholesterol metabolism under CS. Heat maps of mean log2 intensities (left map, warm scale) and corresponding log2 fold change (right map, cool scale) in CS versus REP groups (*n* = 6) of proteins involved in oxidative phosphorylation (**A**) and cholesterol catabolism (**B**). Scale bars are applicable across all corresponding maps.

Mycobacteria are obligate aerobes with the ability to energetically adapt to variable environments and sustain metabolism in the absence of growth (80). Unsurprisingly, most proteins involved in oxidative phosphorylation were unchanged in CS (Fig. 7A). Of the differentially abundant proteins, most were moderately more abundant in CS compared to REP. The most prominent complex-wide difference was in the type I NADH dehydrogenase (NDH-1). This complex is the more energy efficient of two NADH dehydrogenases in *Mab* (80). Five of the identified NDH-1 proteins were significantly more abundant in CS, including NuoG (1.7-fold), NuoH (3.5-fold), NuoM (2.2-fold), and two were only identified in CS (NuoF and NuoN). No NDH-1 proteins were less abundant in CS compared to REP. The overall increase in NDH-1 subunits under CS suggests that *Mab* implements a mechanism of energy conservation under CS. This result agrees with previous reports of transcriptional upregulation of the NDH-1 operon in *Mab* in response to artificial sputum (13) and in *M. smegmatis* under carbon-limited growth (81). We also previously identified similar increased abundance of NDH-1 proteins in *Mtb* grown under CS (17).

Several established anti-mycobacterial drugs target oxidative phosphorylation, and energy production remains an attractive target for novel drug development. The targets of clofazimine, type 2 NADH dehydrogenase (Ndh, MAB_2429c) (82), and bedaquiline, ATP synthase subunit c (AtpE, MAB_1448) (83), were unchanged in CS relative to REP (Table S4). The anti-TB compound GaMF1 targets the γ subunit (AtpG, MAB_1452) (84), which was also unchanged with CS.

#### Cholesterol catabolism

Cholesterol is a primary source of energy for mycobacteria during infection (85). Specifically, in *Mab*, genes involved in cholesterol catabolism were upregulated in a macrophage infection model (32), and a cholesterol catabolism mutant strain (Δ*hsaD*) was unable to replicate in macrophages (86). These findings suggest that *Mab* depends on the ability to break down cholesterol to survive intracellularly. We observed several changes in cholesterol catabolism in response to CS (Fig. 7B). While two enzymes involved in side-chain degradation were absent in CS (ChsE5 and Cyp125), all significantly different A/B ring cleavage enzymes were more abundant in CS. This included HsaD, whose gene is located separate from the *hsaACB* operon uniquely in *Mab* (86). KstR, the TetR family repressor that regulates transcription of both side-chain and A/B ring degradation enzymes (87), was also more abundant in CS (1.9-fold), further supporting the role of cholesterol catabolism in CS.

### Sulfate metabolism

**Fig 8:**
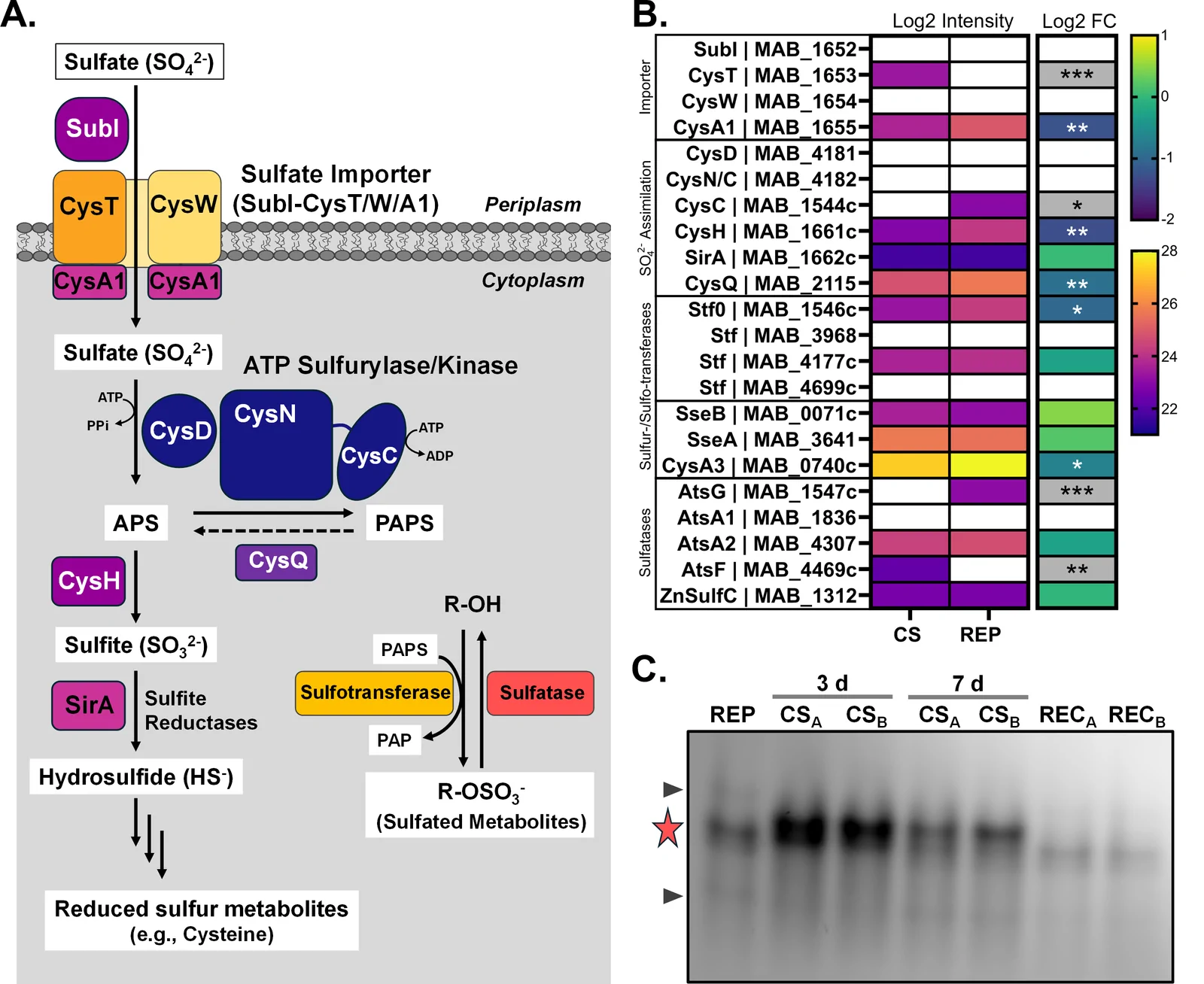
Sulfate metabolism is changed in carbon-starved *Mab*. (**A**) The sulfate assimilation pathway. Inorganic sulfate is acquired through the sulfate importer. It is activated to adenosine-5′-phosphosulfate (APS) by CysD/N/C and converted to 3′-phosphoadenosine-5′-phosphosulfate (PAPS), the universal sulfate donor. PAPS is used by sulfotransferases to sulfate biomolecules. The reverse reaction is catalyzed by sulfatases. Separately, in the reductive path, APS can be converted to sulfite and hydrosulfide. (**B**) Heat map of mean log2 intensities (left map, warm scale) and corresponding log2 fold change (right map, cool scale) of sulfate metabolism-involved proteins in CS versus REP groups (*n* = 6). (**C**) Sulfatase activity detected in gel-resolved lysates of *Mab* incubated with DDAO sulfate. The star highlights sulfatase activity induced in CS. Other sulfatase activity is indicated with arrowheads. REC, recovered from CS.

Mycobacteria acquire sulfate from the environment to produce a variety of sulfated metabolites (88, 89) (Fig. 8). Many of the key enzymes in sulfate metabolism have been characterized in *Mtb* and are conserved in *Mab*. We examined these sulfate metabolic enzymes within our proteomic data to identify changes between CS and REP (Fig. 8B). A multi-protein sulfate importer, named SubI-CysT/W/A1, is used to acquire sulfate from the environment. We identified two of the components, CysA1 (MAB_1655), which was 1.2-fold less abundant in CS, and CysT (MAB_1653), which was only identified in CS. Several enzymes in the sulfate assimilation pathway were less abundant in CS, including CysC (MAB_1544c), CysH (MAB_1661c), and the phosphatase CysQ (MAB_2115). We previously reported that both CysH and CysQ were less abundant in CS in *Mtb* (90). The sulfotransferase MAB_1546c is an ortholog of the *Mtb* enzyme Stf0 (Rv0295c, 74.9% sequence identity), which sulfates trehalose (91). MAB_1546c was 2-fold less abundant in CS. The hydrolysis of sulfate esters is catalyzed by sulfatases, which also changed in response to CS. Specifically, AtsG (MAB_1547c) was identified only in REP, and AtsF (MAB_4469c) was identified only in CS. To summarize, these findings suggest that sulfate acquisition is reduced in CS.

We did a preliminary assessment of *Mab* sulfatase activity in REP and CS conditions. We used a method for detecting active sulfatases in native protein gels using a fluorogenic sulfatase probe, DDAO sulfate, as described previously (92). Briefly, normalized total protein lysates were resolved by native gel electrophoresis, incubated with DDAO sulfate, and imaged. We observed an increase in sulfatase activity in CS samples (Fig. 8C). Upon recovery from CS (REC), activity reverted to lower levels similar to those observed in REP. We have not identified the sulfatases associated with each band, but we speculate that the CS-induced fluorescent band is most likely AtsF because this sulfatase was only identified in CS samples. Given that our data suggest that sulfate acquisition is reduced in CS, it is possible that AtsF activity is used to obtain sulfate directly from endogenous sulfated metabolites.

### Cobalamin biosynthesis

**Fig 9:**
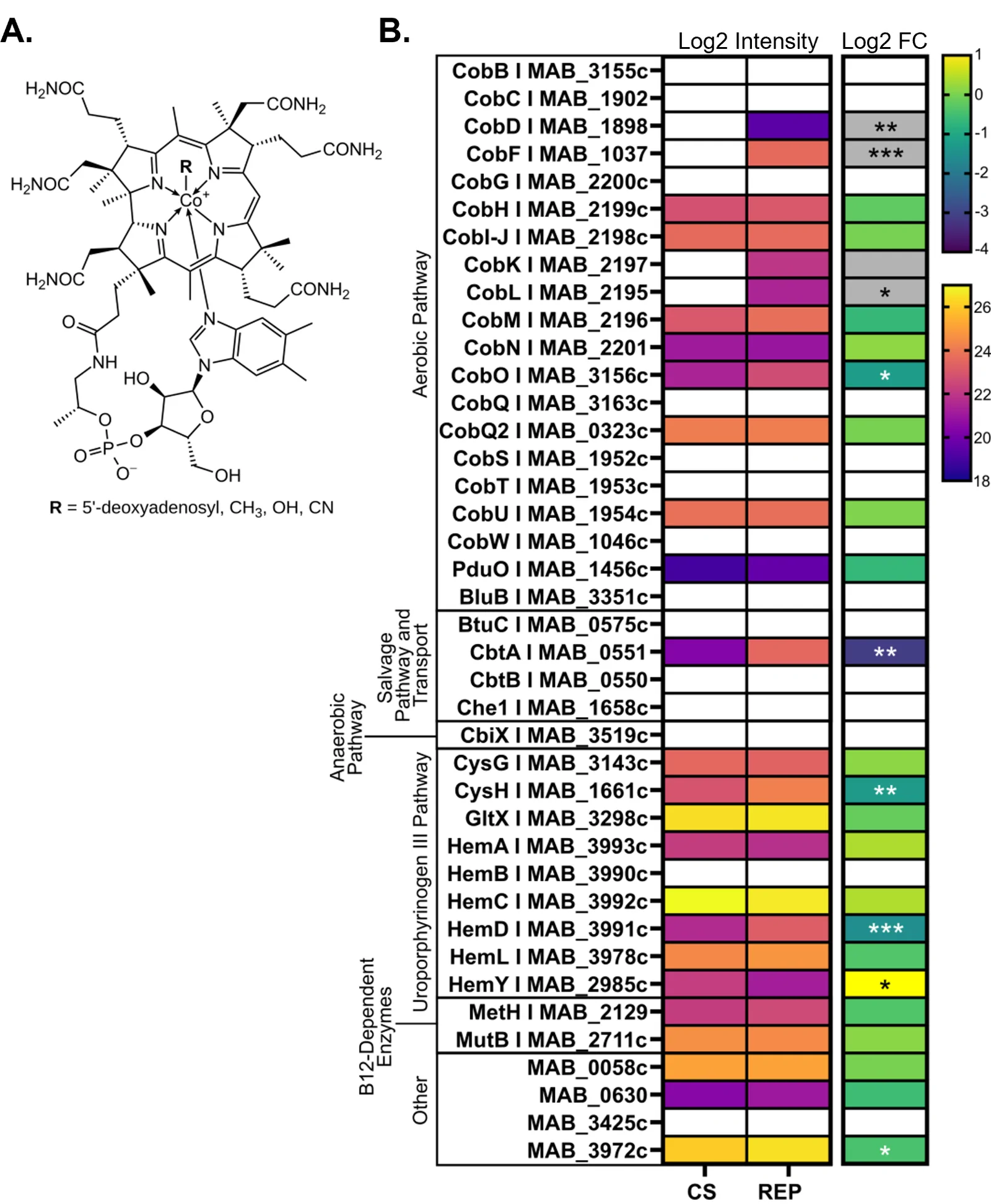
Regulation of *Mab* cobalamin biosynthesis under CS. (**A**) Structure of cobalamin (Wikipedia). (**B**) Heat map of mean log2 intensities (left map, warm scale) and corresponding log2 fold change (right map, cool scale) of cobalamin biosynthesis in CS versus REP groups (*n* = 6).

The cofactor cobalamin, also known as Vitamin B12, has a tetrapyrrole framework that chelates a cobalt ion (Fig. 9). This is a biosynthetically expensive molecule, requiring 30 reactions to make. Many mycobacterial pathogens have the necessary set of enzymes for *de novo* cobalamin biosynthesis (93, 94), and cobalamin has been found in several strains of *Mab* (93). Our data indicate that cobalamin biosynthesis is reduced in CS (Fig. 9B). Specifically, several of the enzymes in the pathway were found only in REP: CobD (MAB_1898), CobF (MAB_1037), CobK (MAB_2197), and CobL (MAB_2195). Two components of the uroporphyrinogen III pathway were >2.5-fold less abundant in CS (CysH and HemD), while HemY (MAB_2985c) was 2-fold more abundant in CS. The cobalt transporter subunit CbtA (MAB_0551) was 8.7-fold less abundant in CS. Two cobalamin-dependent enzymes, MetH (MAB_2129) and MutB (MAB_2711c), were unchanged between REP and CS. Overall, our data indicate that the biosynthetic machinery to make cobalamin is present in both conditions but is more abundant in REP than in CS.

### Iron acquisition, iron-sulfur proteins, and cytochrome P450s

Mycobacteria require iron for growth and infection. The base broth used in both our CS and REP media contains trace metals, including ferric ammonium citrate. Iron acquisition did not appear to be impacted by CS (Table S4). In contrast, differences in iron-sulfur [Fe-S] cofactor biosynthesis, [Fe-S] proteins, and cytochrome P450s were found in CS compared to REP cultures, as described in the next three sections.

#### [Fe-S] cluster biosynthesis

**Fig 10:**
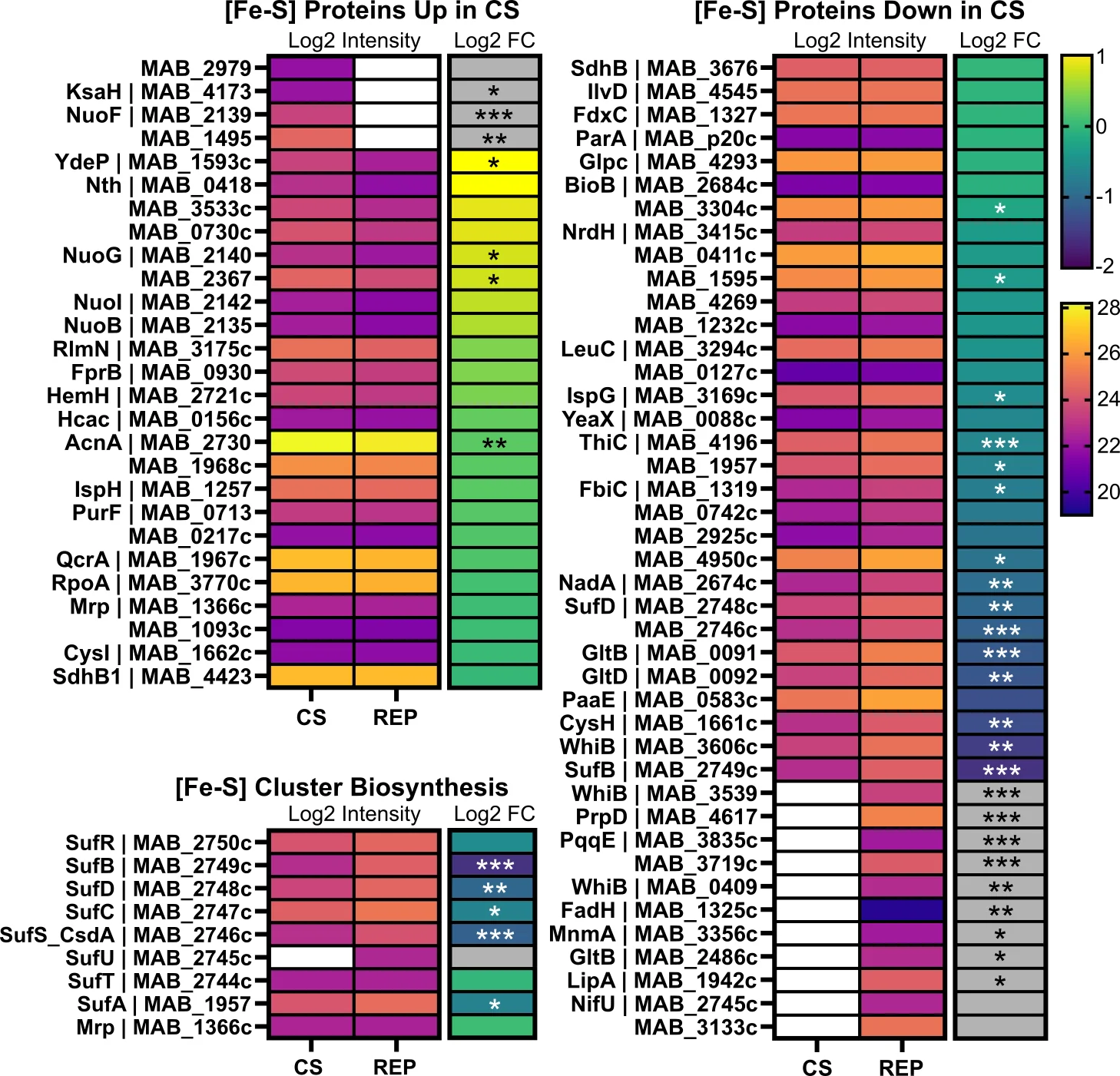
Regulation of *Mab* [Fe-S] cluster biosynthesis enzymes and [Fe-S] proteins under CS. Heat maps of mean log2 intensities (left maps, warm scale) and corresponding log2 fold change (right maps, cool scale) in CS versus REP groups (*n* = 6). Scale bars are applicable across all corresponding maps.

Mycobacteria use the sulfur utilization factor (SUF) machinery to make [Fe-S] clusters (95). The *Mab* SUF operon includes seven genes: sufRBDCSUT (MAB_2750c–MAB_2744c). SufU (MAB_2745c) was found only in REP, while four other components were less abundant in CS: SufB, SufD, SufC, and SufS (Fig. 10). The [Fe-S] cluster delivery protein SufA (MAB_1957) was 1.6-fold less abundant in CS, while two others, Mrp (MAB_1366c) and SufT (MAB_2744c), were unchanged. Our findings suggest that [Fe-S] cluster biosynthesis is downregulated in response to carbon starvation.

#### [Fe-S] cluster proteins

A recent review summarized the roles of mycobacterial [Fe-S] cluster proteins in diverse processes including virulence, persistence, metabolism, and antibiotic resistance (95). The authors identified 126 [Fe-S] cluster proteins encoded by *Mab*; 69 of these were identified in our proteomic samples. Our data indicate that *Mab*’s [Fe-S] cluster proteins are differentially expressed in response to CS. Specifically, 11 [Fe-S] cluster proteins were found only in REP samples, including two WhiB transcriptional regulators (MAB_3539 and MAB_0409). A third WhiB regulator, MAB_3606, was 2.8-fold less abundant in CS. On the other hand, four [Fe-S] cluster proteins were only found in CS (i.e., KsaH, NuoF, MAB_1495, and MsrB). KsaH (MAB_4173) is involved in cholesterol metabolism, and NuoF (MAB_2139) is involved in energy production (Fig. 7). MAB_1495 is a probable oxidoreductase. Finally, MsrB (MAB_2979) is a peptide-methionine (R)-S-oxide reductase.

### Cytochrome P450s

**Fig 11:**
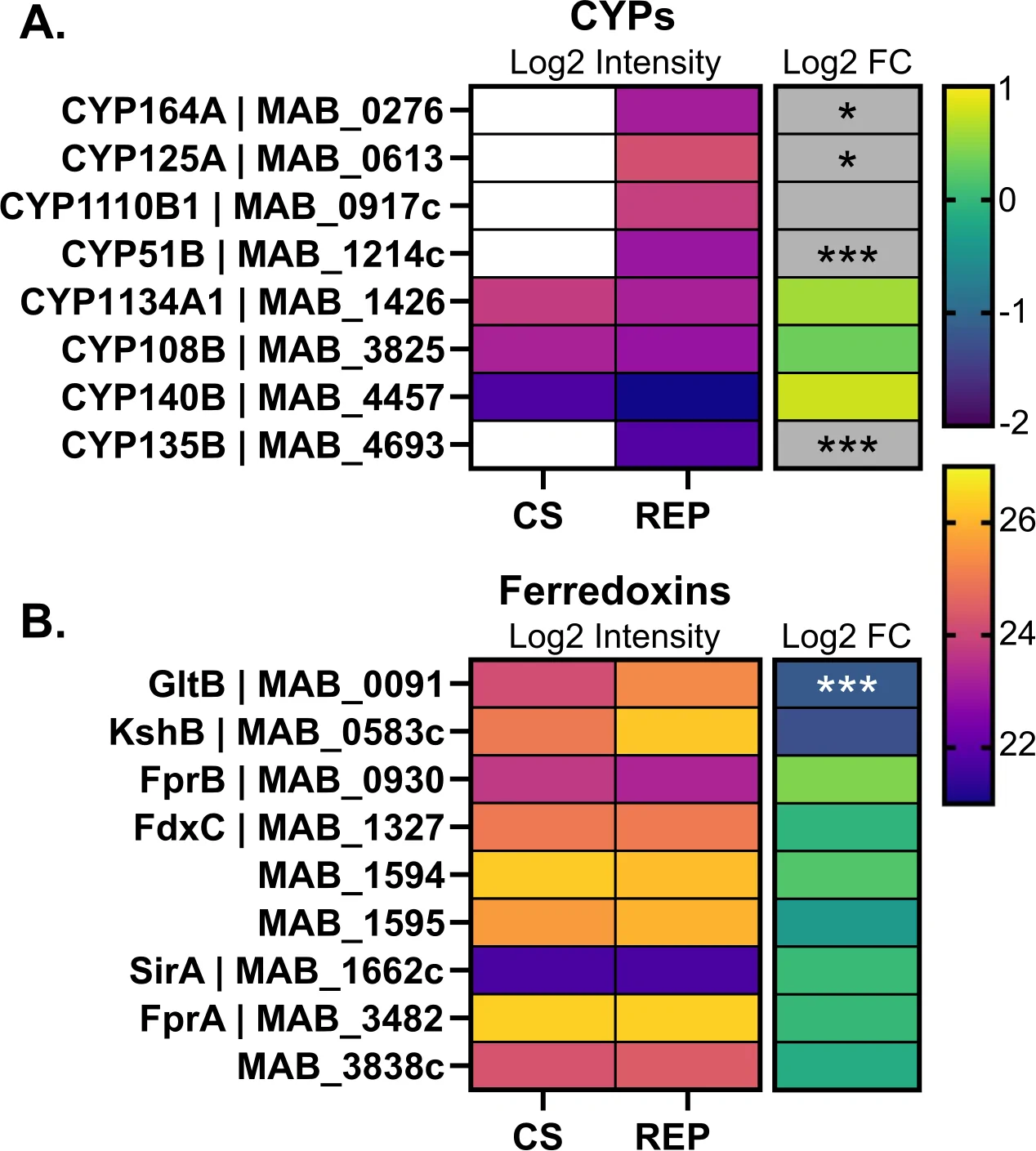
Regulation of *Mab* cytochrome P450s and ferredoxins under CS. Heat map of mean log2 intensities (left map, warm scale) and corresponding log2 fold change (right map, cool scale) of CYPs (**A**) and ferredoxins (**B**) in CS versus REP groups (*n* = 6). Scale bars are applicable across all corresponding maps.

Cytochrome P450s (CYPs) are heme-dependent monooxygenases and potential drug targets. The genome of *Mab* encodes 25 CYPs (96, 97). A complete list of *Mab* CYPs is provided in the Table S4, and they were named according to subfamily, as described (98). We identified eight CYPs in our proteomic samples (Fig. 11). The best described is CYP51, an ancient CYP found across species and kingdoms. In *Mtb*, CYP51 (Rv0764c) is a sterol demethylase (99). The corresponding CYP51B in *Mab* (MAB_1214c) was identified only in REP samples. In our data set, four additional CYPs were identified only in REP samples: CYP164A (MAB_0276), Cyp125A (MAB_0613), CYP1110B1 (MAB_0917c), and CYP135B (MAB_4693). *Mtb*’s CYP125A (Rv3545c) plays a key role in cholesterol metabolism and survival *in vivo* (100, 101), while CYP135B1 may detoxify the anti-TB drug SQ109 (102). The functional consequence of the decreased abundance of these enzymes under CS in *Mab* is unclear. We identified three CYPs that were not significantly changed between REP and CS, namely Cyp1134A1 (MAB_1426), Cyp108B (MAB_3825), and Cyp140B (MAB_4457). We identified nine putative redox partners (e.g., ferredoxins), and all but one were unchanged between REP and CS (Fig. 11B).

### Proteomic response to carbon starvation in *Mab* compared to *Mtb*

We recently described the global proteomic changes in a double auxotrophic strain of *Mtb* (mc^2^6020, Δ*lysA* and Δ*panCD*) under CS (90). *Mtb* is a slow-replicating mycobacterium; therefore, it was subjected to CS for 5 weeks instead of 3 days. Otherwise, the culture conditions were identical to those used in the present study, enabling us to compare findings across species. Both *Mtb* and *Mab* substantially change their proteome in response to CS, with a third of identified proteins altered in abundance or presence in both species.

We compared some of our key findings from *Mab* with those from *Mtb* (Table S5). One notable contrast was that the DosR/S regulon was activated in response to CS in *Mab*, but not in *Mtb*. The abundance of *Mtb* DosR (Rv3133c) and DosT (Rv2027c) was unchanged under CS, while DosS (Rv3132c) was identified in REP samples alone. In both pathogens, the DosR/S regulon is used to adapt to hypoxia (12, 103, 104), suggesting that the discordance between species is specifically associated with CS and not a generalized stress response.

As noted above, the T7SS Esx3 appears to be more relevant to *Mab* during active growth. Our *Mtb* data suggest a similar finding. In *Mtb*, we identified two Esx3 components, EsxG (Rv0287) and PPE4 (Rv0286), in REP alone. Two others were more abundant in REP: EccE3 (Rv0292) and EccA3 (Rv0282). Only one Esx4 protein was identified in *Mtb* (Rv1797), preventing a comparison of that system.

The most striking finding across *Mab* and *Mtb* regarding cell wall regulation under CS was reduced D,D-carboxypeptidase abundance in CS. In both species, DacB1 and DacB2 were among the most repressed proteins in CS. These enzymes make the tetrapeptide substrate used by LDTs to form 3→3 PG crosslinks. Therefore, we speculate that both species have fewer 3→3 crosslinks under CS, although this measurement has not yet been reported in the literature. Two PG endopeptidases (i.e., RipA and RipC) and two other cell division proteins (i.e., FtsZ and Ami2/CwlM) were more abundant in REP in both species. Additionally, mycolic acid biosynthesis appeared reduced to a similar extent under CS in *Mab* and *Mtb*.

Analysis of energy metabolism proteins also highlighted similarities between the *Mab* and *Mtb* responses to CS. Both species appeared to favor NDH-1, with the majority of NDH-1 proteins more abundant in CS. As the more energy-efficient NADH dehydrogenase in mycobacteria (80), it is probable that the enrichment of NDH-1 serves as an energy-saving mechanism in both species under starvation conditions. On the other hand, little overlap of abundance changes in cholesterol catabolism proteins was observed between *Mab* and *Mtb*. A key regulator of cholesterol catabolism, KstR, was more abundant under CS in *Mab* but less abundant in *Mtb*.

As discussed, iron uptake was largely unchanged in CS in *Mab*. In contrast, proteins involved in siderophore biosynthesis (105) and uptake (106) were absent in CS in *Mtb*. On the other hand, [Fe-S] cluster biosynthesis proteins were overall repressed in CS in both *Mab* and *Mtb*. There were some shared changes in [Fe-S] cluster proteins, including lowered abundance for eight proteins in CS and absence of WhiB and FadH. Less agreement between species was observed among [Fe-S] cluster proteins that were more abundant under CS in *Mab*.

A caveat of this comparative analysis is that we did not exhaustively compare every proteomic change found in *Mab* versus *Mtb* in response to CS. Furthermore, comparative genomic analysis determined that *Mab* is a distinct organism that is only distantly related to other mycobacteria, including *Mtb* (1). This makes comparison more challenging. It is likely that there are some interesting differences that remain to be described.

### Conclusions

The ability to enter and maintain a non-replicating state is critical for *Mab* to maintain chronic infections in humans. It is therefore crucial to understand this physiological state. Several *in vitro* models mimicking microenvironmental conditions within the host induce a non-replicating state in *Mab*. We recently described a simple and accessible model based on carbon starvation, which induces a non-replicating but viable state (20). Herein, we provide the first detailed analysis of the proteomic changes associated with CS, broadening our understanding of the physiology of persistent *Mab*.

We found that *Mab* uses two-component signal transduction systems to respond to CS. Similar to the hypoxic response (12, 31), the stress response regulators DosRS and seven corresponding downstream proteins were more abundant under CS. We also found widespread alterations in enzymes responsible for secretion, nutrient biosynthesis, nutrient acquisition, and metabolism. Consistent with other mycobacterial persistence models, we observed a shift toward energy conservation within oxidative phosphorylation under CS, with an increased abundance of the energy-efficient type I NADH dehydrogenase complex.

Our data support that the non-replicating state is maintained by altering the cell envelope, including PG biosynthesis and degradation. Numerous changes in the cell envelope machinery were noted, including significant changes in the GPL locus. We confirmed the effect of these proteomic changes by demonstrating that two classes of glycolipids, PIMs and GPLs, underwent substantial changes in CS. The outcome of these changes is unclear but could be profound, considering the cell envelope is both a barrier and the first point of contact with the host. A limitation of our lipid analysis is that we did not closely examine mycolic acids.

Overall, the detailed description of *Mab*’s proteomic remodeling in CS broadens our understanding of the molecular mechanisms and characteristics of *Mab* non-replicating persistence. Our analysis also enabled the identification of several key similarities and differences in how *Mab* responds to CS compared to *Mtb*. The data provided here will serve as a rich resource for further work using *in vitro* persistence models.

## MATERIALS AND METHODS

See the Supplemental material for details on materials, experimental design, culture conditions, mass spectrometry, data analysis, lipid extraction, and detection of sulfatase activity.

## Data Availability

Proteomics data were deposited in MassIVE under accession code MSV000101321.

## SUPPLEMENTAL MATERIAL

Supplemental material msystems.00564-26-s0001.pdf Supplemental materials and methods; Figures S1 and S2.

Table S1 msystems.00564-26-s0002.xlsx Protein statistics from global proteomic analysis.

Table S2 msystems.00564-26-s0003.xlsx DEP between CS and REP samples.

Table S3 msystems.00564-26-s0004.xlsx Determination of Mab Mtb orthologues by bioinformatic analysis.

Table S4 msystems.00564-26-s0005.xlsx DEPs by category.

Table S5 msystems.00564-26-s0006.xlsx Proteomics comparison of Mab versus Mtb.

ASM does not own the copyrights to Supplemental Material that may be linked to, or accessed through, an article. The authors have granted ASM a non-exclusive, world-wide license to publish the Supplemental Material files. Please contact the corresponding author directly for reuse.
