# Supplementary material for: Carbon starvation of Mycobacterium abscessus induces a non-replicating state with extensive proteomic remodeling
Source: mSystems. 2026 Sep 23;11(10):e00564-26. doi: 10.1128/msystems.00564-26 (PMC13712860; doi:10.1128/msystems.00564-26)
Supplement: Supplementary file 1 [file msystems.00564-26-s0001.pdf]

Electronic supporting information for:

Carbon starvation of *Mycobacterium abscessus* induces a non-replicating state with extensive proteomic remodeling

Kaylyn L. Devlin[1], Gyanu Lamichhane[2], William C. Nelson[3], Vivian S. Lin[3,4], Kimberly E. Beatty[1*]

1. Department of Chemical Physiology and Biochemistry, Oregon Health & Science University, Portland, Oregon 97239
2. Division of Infectious Diseases, School of Medicine, Johns Hopkins University, Baltimore, Maryland 21231
3. Biological Sciences Division, Pacific Northwest National Laboratory, Richland, Washington 99354
4. Institute of Biological Chemistry, Washington State University, Pullman, Washington 99164

* Corresponding author: 503-494-9359, beattyk@ohsu.edu

Materials and methods

Solvents and chemicals

ACS grade solvents were used as received. Chemicals were purchased from Sigma-Aldrich, Fisher Scientific, or Chem-Impex and used as received. Lipid analysis by thin-layer chromatography (TLC) was done on glass-backed silica gel plates (Merck HP-TLC Silica gel 60 Å, 5-6 μM particle size).

Experimental design and statistical rationale

A total of 12 samples were analyzed in this study, comprised of six biological replicates in two distinctly cultured groups: Replicating (REP; n = 6) and Carbon Starvation (CS; n = 6). The number of replicates was determined for sufficient power in quantitation and statistical analyses. Samples were analyzed by liquid chromatography tandem mass spectrometry (LC-MS/MS) in randomized order.

Mycobacterial culture conditions

Mycobacterium abscessus (*Mab*) ATCC 19977 (subsp. *abscessus*) and a clinical isolate M9510[1] (subsp. *massiliense*) were obtained from the G. Lamichhane laboratory (Johns Hopkins University). Strains were handled as BSL-2 pathogens under OHSU-approved biosafety protocols. All bacterial manipulation was done within a biosafety cabinet to minimize the potential for exposure to the pathogen.

Bacteria were thawed from frozen stocks stored at -80 °C in 30% glycerol. *Mab* ATCC 19977 was cultured in LGM1 medium (Middlebrook 7H9 broth [BD Difco], 0.5% glycerol [Fisher, molecular biology grade], 0.05% Tween 80 [Sigma], 0.5% bovine serum albumin fraction V [RPI, molecular biology grade], 11.1 mM D-dextrose [Sigma], 13.8 mM sodium chloride [Sigma]). Cultures were grown at 37 °C with 100 rpm in aerated polycarbonate shake flasks with a 0.2 μm filter cap (Weaton #WPFPC0500S).

Culture conditions to induce a non-replicating state via carbon starvation (CS) were adapted from previously reported methods[2,3]. Samples for proteomic analysis were generated from exponential phase *Mab* ATCC 19977 grown in LGM1. Cells were washed twice with PBS and diluted to an optical density 600 (OD_{600}) of 0.2 in CS medium (7H9 broth, 0.05% tyloxapol [Sigma]). Cultures (n = 6, 300 mL) were grown standing at 37 °C in 1 L plug-sealed bottles

(Corning #430195) for 72 hrs. Matched replicating cultures (REP) were simultaneously prepared from the same washed cell stock. Cells were diluted to an OD_{600} of 0.2 in LGM1-Tx (supplemented with 0.05% tyloxapol instead of Tween 80) medium. REP cultures (n = 6, 200 mL) were grown shaking (100 rpm, 37 °C) in aerated 500 mL shake flasks until an OD_{600} of ~0.4 was reached. Cultures were harvested through centrifugation (5 min, 4000 xg, 4 °C), washed twice with PBS, and stored at -30 °C in PBS until lysis.

CS cultures for lipid analysis were grown as above but were maintained in CS medium for 3-7 days. REP cultures for lipid analysis were grown in LGM1-Tx to an OD_{600} of 0.4–1.0. To analyze recovery from CS (REC), carbon starved cultures were diluted 1:10 or 1:50 in LGM1-Tx and grown for 1 day before harvest (OD_{600} = 0.2 – 0.5).

Lysate preparation
Cells were lysed following a protocol adapted from previously described methods[4]. Frozen cell pellets were thawed on ice. Cells were lysed in 1 mL of PBS by mechanical disruption with a MiniLys Beadbeater (Bertin Technologies) at 5,000 rpm (3x 45 s, cooling between on ice for 180 s) using 0.1 mm zirconia/silica beads (BioSpec Products). Beads and cell debris were pelleted by centrifugation (18,000 xg, 5 min, 4 °C) and the clarified supernatant was collected. Beads and cell debris were then incubated in 1 mL of 1% n-dodecyl-D-β-maltoside (Chem-Impex #21950, CAS 69227-93-6) in PBS (PBS-DM) for 30 min on ice. The second supernatant was collected after centrifugation and added to the clarified supernatant, resulting in a final lysate in 0.5% PBS-DM. Final lysates were centrifuged to remove residual beads (18,000 xg, 5 min, 4 °C) and filtered twice through 0.2 µm PVDF membrane filters (13 mm, Pall) to sterilize. A bicinchoninic acid (BCA) assay (Pierce) was used to quantify the total protein concentration of all lysates.

Mass spectrometry sample preparation
Fresh lysates from CS and REP samples (150 µg total protein) were digested with proteomics-grade trypsin (Promega) overnight at RT with end-over-end rotation. Solid Phase Extraction (SPE) C18 columns (50 mg bed wt., Millipore-Sigma Supelco Discovery DSC-18) were conditioned with methanol (3 mL, under vacuum, Fisher) and rinsed with acidified water (2 mL, 0.1% trifluoroacetic acid [TFA], Fisher). The peptides from the digestion were applied to the SPE columns and slowly allowed to pass through the column (≤ 1 mL/min). Samples were washed with 4 mL of 95:5 H_2O:acetonitrile (ACN, Fisher) with 0.1% TFA. Columns were allowed to go to dryness, and column tips were wiped to remove any residue. Peptides were slowly eluted from columns with 20:80 H_2O:ACN with 0.1% TFA (1 mL), under vacuum. The samples were dried in a SpeedVac concentrator and resuspended in HPLC-grade water (50 µL). Peptide concentrations were quantified via BCA assay. Samples were normalized to 50 µL of 0.1 µg/µL of total peptide, and 30 µL of each sample was transferred to vials (MicroSolv 2 mL vials with AQ Brand deactivated glass low volume inserts) for MS analysis.

Liquid chromatography tandem mass spectrometry
LC-MS/MS analysis of global proteomics samples was conducted using a Waters nanoAcquity ultra performance liquid chromatography (UPLC) system connected to a Q Exactive Plus Orbitrap mass spectrometer (Thermo Scientific). Samples were loaded into a precolumn (150 µm i.d., 4 cm length, packed in-lab with Jupiter C18 packing material, 300 Å pore size, 5 µm particle size; Phenomenex) using mobile phase A (0.1% formic acid in water). The separation was carried out using a self pack NanoLC column (CoAnn Technologies, 75 µm i.d., 30-cm column) with Waters BEH C18 packing material, 130-Å pore size, 1.7 µm particle size (Waters Corporation). Separations were performed at a flow rate of 200 nL/min using a 120 min gradient of 1-75% mobile phase B (ACN + 0.1% formic acid). To prevent carryover, the column

was washed with 95-50% mobile phase B for 20 min and equilibrated with 1% mobile phase B for 30 min before the next sample injection.

The mass spectrometer source was set at 2.2 kV, and the ion transfer capillary was heated to 300 °C. The data-dependent acquisition mode was employed to automatically trigger the precursor scan and the MS/MS scans. The MS1 spectra were collected at a scan range of 300-1800 m/z, a resolution of 70,000, an automatic gain control (AGC) target of 3×10^6, and a maximum ion injection time of 20 ms. For MS2, the top 12 most intense precursors were isolated with a window of 1.5 m/z and fragmented by higher-energy collisional dissociation (HCD) with a normalized collision energy at 30%. The Orbitrap was used to collect MS/MS spectra at a resolution of 17,500, a maximum AGC target of 1×10^5, and maximum ion injection time of 50 ms. Each parent ion was fragmented once before being dynamically excluded for 30 s.

Analysis of mass spectrometry data

Proteomics data were deposited in MassIVE under accession code: MSV000101321 (https://massive.ucsd.edu/ProteoSAFe/dataset.jsp?task=e82dfd23322b4b9690108cd5d8ebb2b6).

MS/MS Automated Selected Ion Chromatogram (MASIC) generator was used to generate selected ion chromatograms (SICs) for all of the parent ions chosen for fragmentation in the LC-MS/MS data[5]. MSGF+ software (v2024.03.26)[6] was then used to perform peptide searches against the *M. abscessus* protein database (UniProt taxon ID 561007, downloaded on 2024-01-15) and 16 common contaminant sequences (porcine and bovine trypsin, chymotrypsinogen, human and bovine albumin, and some keratins). Searches were performed with the following parameters: partially and fully tryptic peptides; parent ion tolerance of 20 ppm; methionine oxidation (+15.9949 Da) as a dynamic modification. MSGF+ first hits were exported, and peptides were filtered using a spectral probability value of 1.56×10^{-8} corresponding to an estimated 1% false discovery rate (FDR) as calculated previously[7] using the target-decoy approach[8] for *M. tuberculosis* global proteomics data.

Global proteomics analyses were performed using the Multi-Omics Analysis Portal and the pmartR package[9]. Potential sample outliers were assessed by a robust Mahalanobis distance (rMd) using the rMd squared values associated with the peptide abundances vector (rMd-PAV), which was applied with all default metrics for proteomics data (correlation, kurtosis, MAD, skewness, and proportion missing). No sample was identified as an outlier, and all samples were included in analyses. Peptide data were log2 transformed and median normalized. Redundant peptides (those mapping to more than one protein) and peptides found in less than two samples were removed before protein rollup. Peptide ion intensities were rolled up to the protein level using the Rrollup method[10] with median centering. Proteins mapped by less than two peptides were removed. Proteins had to be identified in at least three samples of one group to be included in analysis. Statistical differences in protein abundance between groups was assessed via analysis of variance (ANOVA) and independence of missingness (IMD, G-test)[11]. In cases when a protein was observed in at least two samples in each group, it was considered significantly different if the mean log2 abundance between groups had a $P \leq 0.05$ by ANOVA. In cases when a protein was observed in less than two samples in one group, the number of observed values between groups was considered statistically different if $P \leq 0.05$ by IMD.

Protein functional classification and ortholog analysis

Functional classification of *Mab* proteins was done using UniProt[12] annotation (taxon ID 561007,

downloaded 2024-01-15). Ortholog mapping compared the *Mab* 19977 proteome to the *Mycobacterium tuberculosis* H37RV proteome (UniProt taxon ID 83332, downloaded 2024-06-27). Protein family assignments were calculated using five methods: **1)** Markov clustering of DIAMOND (v 2.1.13)[13] blastp all versus all search results using MCL (v 12-068)[14]; **2)** OrthoFinder (v 2.5.4)[15]; **3)** MMSEQS2 (v 13.45111)[16] easy-cluster workflow with the cluster-reassign option; **4)** reciprocal best hit (RBH) analysis of the DIAMOND all versus all results using two standard deviations from the RBH mean amino acid identity as a cutoff and MultiParanoid[17] to cluster results; and **5)** hmmer (v 3.4) (https://hmmer.org) search against the 2983 'equivalog' models of TIGRfam (v 15.0)[18] (now part of NCBI's PGAP hmm library[19]). These data were supplemented with gene synteny information determined by examining the two upstream and downstream genes of each RBH pair for RBH relationships (*code available upon request* from W. Nelson). Final integrated family assignment was accomplished by prioritizing families with one member per genome and reporting all individual method family assignments in common for all integrated family members (*code available upon request* from W. Nelson). In cases where multiple proteins in a genome had equivalent scoring for a given integrated family, all proteins were included.

Polar lipid extraction and TLC analysis

Mab 19977 was cultured under REP and CS conditions as described above, except that CS was 7 days. Cells (50 mL volume) were pelleted (2500 xg, 5 min, 4 °C), washed once with PBS, and stored at -30 °C until use. The polar and apolar lipid extracts were obtained, as described by Besra[20]. Cell pellets were heat-killed at 65 °C for 1 hr before apolar extraction. Briefly, 600 µL/sample of 100:10 (methanol:0.3% $NaCl_{aq}$) was added to cells, followed by 300 µL of petroleum ether. Samples were vortexed and extracted for 15 min at RT. Samples were pelleted (3500 xg, 10 min, RT) and the upper layer (apolar lipids) was transferred to a new tube. The lower layer was extracted a second time with petroleum ether (600 µL) for 10-30 min and pelleted. Again, the upper layer was transferred to the same tube. The combined apolar lipid extracts were evaporated under nitrogen gas and stored dry (-20 °C).

Next, polar lipids were extracted into 800 µL chloroform/methanol/(0.3% $NaCl_{aq}$) [9:10:3] overnight at 4 °C. Samples were vortexed and pelleted (3500 xg, RT). The solvent extract was placed in a clean tube, and the residue was extracted again with 500 µL chloroform/methanol/(0.3% $NaCl_{aq}$) [5:10:4] for 30 min. The lower layer was combined with the prior extract (the upper layer was discarded). The combined organic extracts were mixed with 1.2 mL chloroform and 1.2 mL 0.3% $NaCl_{aq}$. Samples were centrifuged (3500 xg, 5 min, RT) to obtain the lower organic layer (polar lipid extract); the upper (aqueous layer) was discarded. The combined polar extracts were dried under nitrogen gas. The polar lipids were resuspended in chloroform/methanol (2:1) in a volume normalized to the OD_{600} of the original culture (OD_{600} x 150 µL).

Normalized polar extracts were resolved on a 10x10 cm HP-TLC Silica60 (Merck) plates. For glycopeptidolipid analysis, 2-6 µL were spotted and then resolved in chloroform:methanol:water (90:10:1). For phosphatidylinositol mannoside analysis, samples were resolved in chloroform:methanol:13 M ammonia/(0.1 M ammonium acetate)/water [180:140:9:9:23], as described[21]. TLC plates were dip-stained in orcinol (0.2% orcinol in 20% sulfuric acid) and heated to reveal glycolipids. Plates were imaged on a flatbed scanner (600 dpi) for analysis.

Total lipid extraction

REP, CS, and recovered (REC) cultures from *Mab* 19977 and *Mab* M9510 were prepared for

total lipid extraction, as described[20]. Briefly, thawed pellets (from 50 mL cultures) were extracted in chloroform/methanol (2:1). Samples were heated at 50 °C for 1 hr, and the organic layer was obtained after centrifugation (3500 xg, 10 min, RT). The pellet was extracted a second time in chloroform/methanol (2:1). The combined extracts were dried under nitrogen and then dissolved in chloroform/methanol/0.3% $NaCl_{aq}$ (8:4:2). After 10 min, the sample was centrifuged to collect the lower (organic) layer. This was dried under nitrogen to obtain the total lipid extract, which was resuspended in chloroform:methanol (2:1) in a volume normalized to the OD_{600} of the original culture (OD_{600} X 150 μL). Total lipids were analyzed by HP-TLC as described above.

In-gel sulfatase activity detection

DDAO-sulfate was synthesized as previously described[22]. *Mab* lysates were prepared in PBS-DM, as described above. Lysates were concentrated and buffer exchanged into 50 mM Tris (pH 8 at 4 °C), 150 mM NaCl using Microsep 10 kDa MWCO filters (Pall). Protein was quantified by BCA. Normalized protein was prepared in the same buffer plus 1% sarkosyl and 11 μg/sample was resolved by Native PAGE (10% Tris-Cl gel in 1X Tris-Glycine buffer, BioRad). The gel was soaked in a solution of 15 μM DDAO-sulfate for 10-15 min and then imaged on a Typhoon scanner. DDAO was detected using a 633 nm excitation laser with a 670 nm (bp 30) emission filter. The resulting images were analyzed in ImageJ.

Supplementary figures

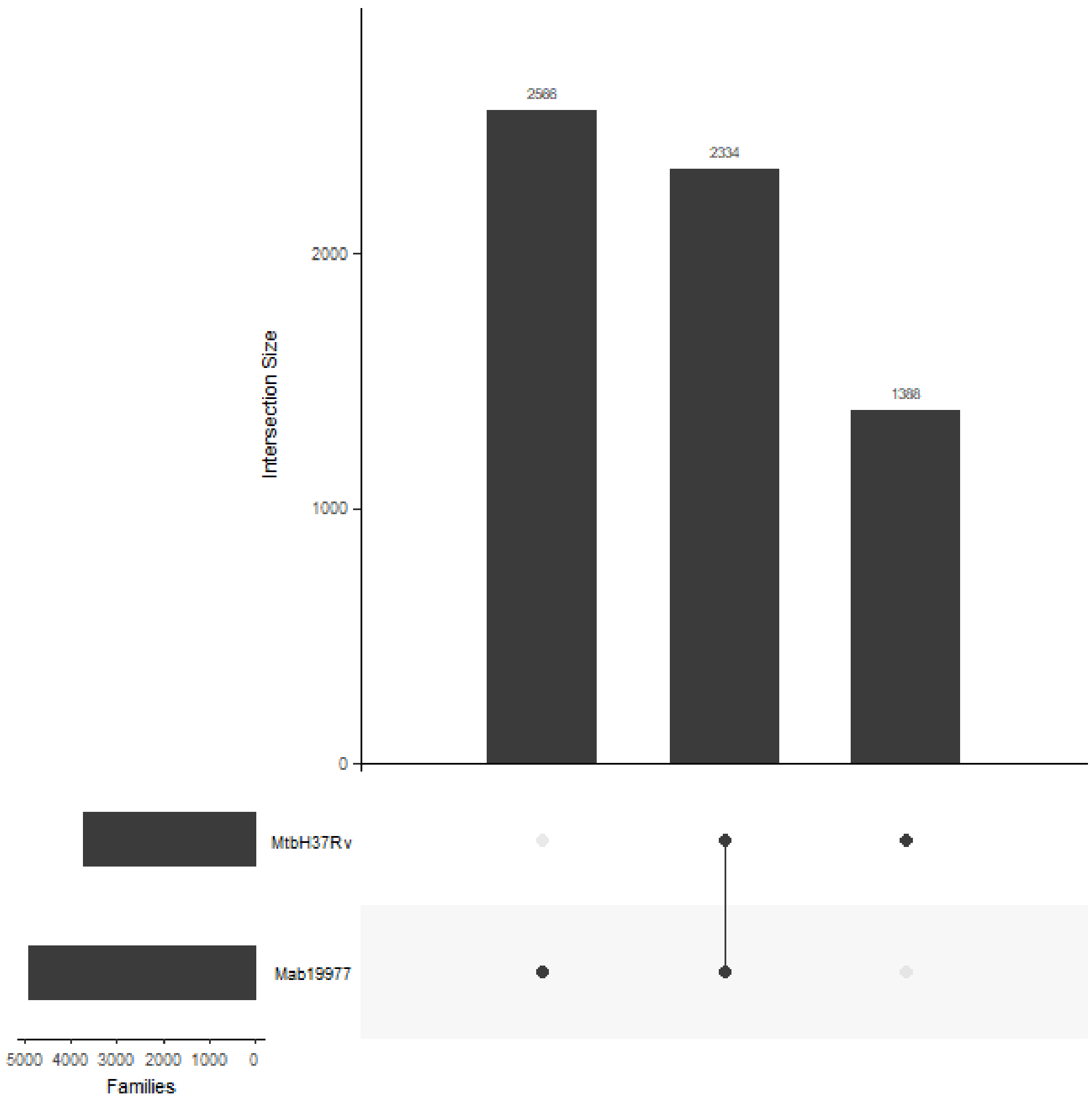

Figure S1. Ortholog determination across *Mab* 19977 and *Mtb* H37Rv. Upset plot showing intersection of ortholog families between organisms. Ortholog families can contain more than one member per genome.

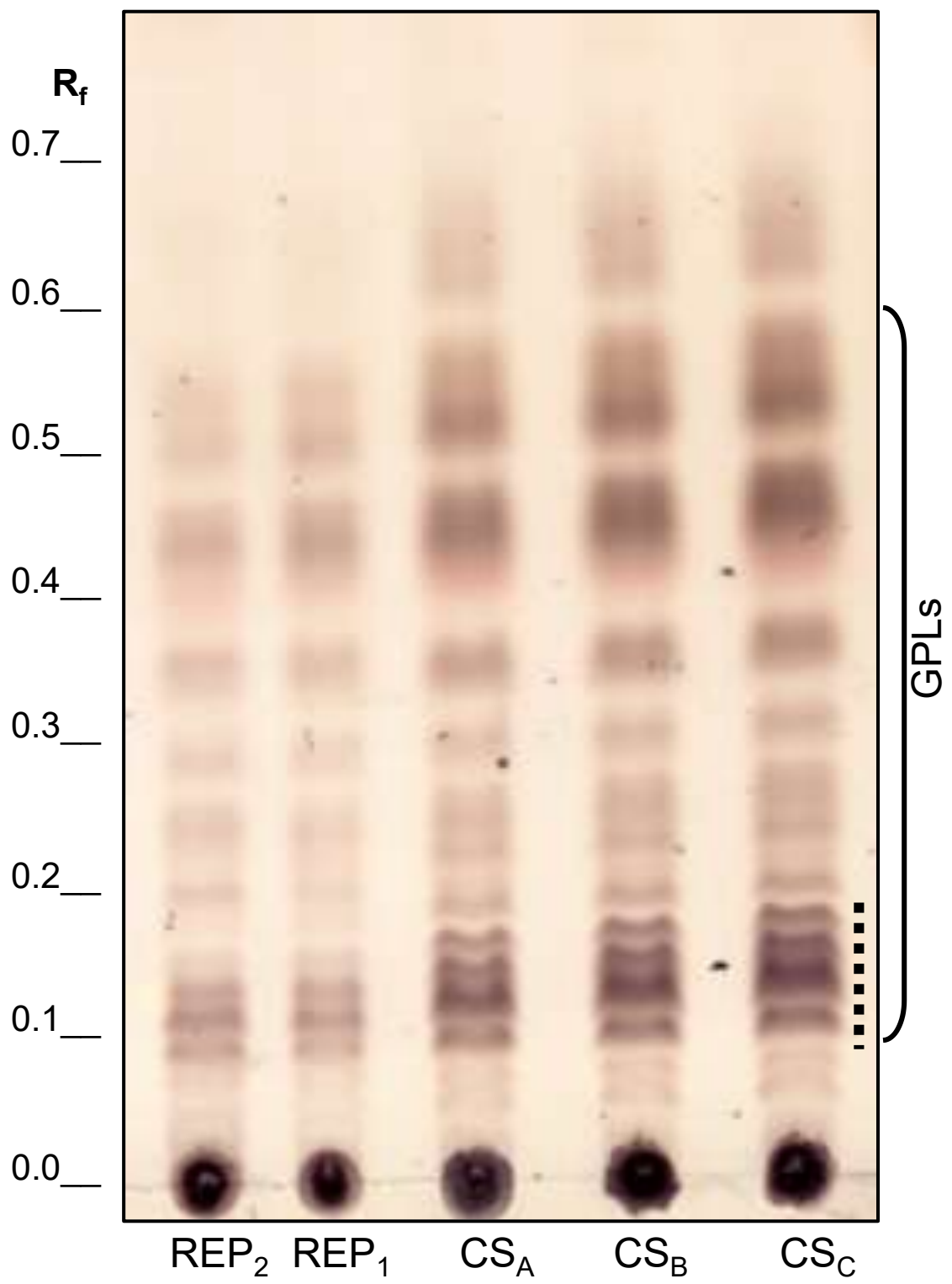

Figure S2. TLC analysis of total polar lipid extracts from *Mab* 19977. Lipids were resolved in chloroform/methanol/water [90:10:1, v:v:v] and glycopeptidolipids were visualized by orcinol staining. Putative GPLs are indicated by a bracket, with the region (R_F = ~0.1-0.2) marked by a dashed line predicted to correspond to GPL3.

References

1 Story-Roller, E., Galanis, C. & Lamichhane, G. beta-Lactam Combinations That Exhibit Synergy against Mycobacteroides abscessus Clinical Isolates. *Antimicrob Agents Chemother* **65** (2021). https://doi.org:10.1128/AAC.02545-20

2 Devlin, K. L., Hutchinson, E., Leach, D. T., Gorham, L. J., Nelson, W. C., Lamichhane, G., Lin, V. S. & Beatty, K. E. Chemoproteomic elucidation of beta-lactam drug targets in Mycobacterium abscessus. *bioRxiv* (2025). https://doi.org:10.64898/2025.12.15.694292

3 Grant, S. S., Kawate, T., Nag, P. P., Silvis, M. R., Gordon, K., Stanley, S. A., Kazyanskaya, E., Nietupski, R., Golas, A., Fitzgerald, M., Cho, S., Franzblau, S. G. & Hung, D. T. Identification of novel inhibitors of nonreplicating Mycobacterium tuberculosis using a carbon starvation model. *ACS Chem Biol* **8**, 2224-2234 (2013). https://doi.org:10.1021/cb4004817

4 Devlin, K. L., Hutchinson, E., Dearing, H. N., Levine, S. R., Reid, D. J., Leach, D. T., Griggs, L. H., Lomas, G. X., Gorham, L. J., Wright, A. T., Lamichhane, G., Lin, V. S. & Beatty, K. E. Comprehensive Identification of beta-Lactam Antibiotic Polypharmacology in Mycobacterium tuberculosis. *ACS Infect Dis* **11**, 2422-2433 (2025). https://doi.org:10.1021/acsinfecdis.5c00233

5 Monroe, M. E., Shaw, J. L., Daly, D. S., Adkins, J. N. & Smith, R. D. MASIC: a software program for fast quantitation and flexible visualization of chromatographic profiles from detected LC-MS(/MS) features. *Comput Biol Chem* **32**, 215-217 (2008). https://doi.org:10.1016/j.compbiolchem.2008.02.006

6 Kim, S. & Pevzner, P. A. MS-GF+ makes progress towards a universal database search tool for proteomics. *Nat Commun* **5**, 5277 (2014). https://doi.org:10.1038/ncomms6277

7 Devlin, K. L., Leach, D. T., Stratton, K. G., Lamichhane, G., Lin, V. S. & Beatty, K. E. Proteomic

characterization of Mycobacterium tuberculosis subjected to carbon starvation. *mSystems* **10**, e0153024 (2025). https://doi.org:10.1128/msystems.01530-24
8 Elias, J. E. & Gygi, S. P. Target-decoy search strategy for mass spectrometry-based proteomics. *Methods Mol Biol* **604**, 55-71 (2010). https://doi.org:10.1007/978-1-60761-444-9_5
9 Degnan, D. J., Stratton, K. G., Richardson, R., Claborne, D., Martin, E. A., Johnson, N. A., Leach, D., Webb-Robertson, B. M. & Bramer, L. M. pmartR 2.0: A Quality Control, Visualization, and Statistics Pipeline for Multiple Omics Datatypes. *J Proteome Res* **22**, 570-576 (2023). https://doi.org:10.1021/acs.jproteome.2c00610
10 Polpitiya, A. D., Qian, W.-J., Jaitly, N., Petyuk, V. A., Adkins, J. N., Camp, D. G., II, Anderson, G. A. & Smith, R. D. DAnTE: a statistical tool for quantitative analysis of -omics data. *Bioinformatics* **24**, 1556-1558 (2008). https://doi.org:10.1093/bioinformatics/btn217
11 Webb-Robertson, B.-J. M., McCue, L. A., Waters, K. M., Matzke, M. M., Jacobs, J. M., Metz, T. O., Varnum, S. M. & Pounds, J. G. Combined Statistical Analyses of Peptide Intensities and Peptide Occurrences Improves Identification of Significant Peptides from MS-Based Proteomics Data. *Journal of Proteome Research* **9**, 5748-5756 (2010). https://doi.org:10.1021/pr1005247
12 The UniProt, C. UniProt: the universal protein knowledgebase. *Nucleic Acids Res* **45**, D158-D169 (2017). https://doi.org:10.1093/nar/gkw1099
13 Buchfink, B., Reuter, K. & Drost, H. G. Sensitive protein alignments at tree-of-life scale using DIAMOND. *Nat Methods* **18**, 366-368 (2021). https://doi.org:10.1038/s41592-021-01101-x
14 Van Dongen, S. Graph Clustering Via a Discrete Uncoupling Process. *SIAM J. Matrix Anal. Appl.* **30**, 121-141 (2008). https://doi.org:10.1137/040608635
15 Emms, D. M. & Kelly, S. OrthoFinder: phylogenetic orthology inference for comparative genomics. *Genome Biol* **20**, 238 (2019). https://doi.org:10.1186/s13059-019-1832-y
16 Steinegger, M. & Soding, J. Clustering huge protein sequence sets in linear time. *Nat Commun* **9**, 2542 (2018). https://doi.org:10.1038/s41467-018-04964-5
17 Alexeyenko, A., Tamas, I., Liu, G. & Sonnhammer, E. L. Automatic clustering of orthologs and inparalogs shared by multiple proteomes. *Bioinformatics* **22**, e9-15 (2006). https://doi.org:10.1093/bioinformatics/btl213
18 Haft, D. H., Selengut, J. D. & White, O. The TIGRFAMs database of protein families. *Nucleic Acids Res* **31**, 371-373 (2003). https://doi.org:10.1093/nar/gkg128
19 Tatusova, T., DiCuccio, M., Badretdin, A., Chetvernin, V., Nawrocki, E. P., Zaslavsky, L., Lomsadze, A., Pruitt, K. D., Borodovsky, M. & Ostell, J. NCBI prokaryotic genome annotation pipeline. *Nucleic Acids Res* **44**, 6614-6624 (2016). https://doi.org:10.1093/nar/gkw569
20 Besra, G. S. Preparation of cell-wall fractions from mycobacteria. *Methods Mol Biol* **101**, 91-107 (1998). https://doi.org:10.1385/0-89603-471-2:91
21 Nguyen, P. P., Kado, T., Prithviraj, M., Siegrist, M. S. & Morita, Y. S. Inositol acylation of phosphatidylinositol mannosides: a rapid mass response to membrane fluidization in mycobacteria. *J Lipid Res* **63**, 100262 (2022). https://doi.org:10.1016/j.jlr.2022.100262
22 Beatty, K. E., Williams, M., Carlson, B. L., Swarts, B. M., Warren, R. M., van Helden, P. D. & Bertozzi, C. R. Sulfatase-activated fluorophores for rapid discrimination of mycobacterial species and strains. *Proc Natl Acad Sci U S A* **110**, 12911-12916 (2013). https://doi.org:10.1073/pnas.1222041110
